# Harnessing α-l-fucosidase for *in vivo* cellular senescence imaging†

**DOI:** 10.1039/d1sc02259h

**Published:** 2021-06-25

**Authors:** Seyoung Koo, Miae Won, Hao Li, Won Young Kim, Mingle Li, Chenxu Yan, Amit Sharma, Zhiqian Guo, Wei-Hong Zhu, Jonathan L. Sessler, Jin Yong Lee, Jong Seung Kim

**Affiliations:** Department of Chemistry, Korea University Seoul 02841 Korea jongskim@korea.ac.kr; Department of Chemistry, Sungkyunkwan University Suwon 16419 Korea; Institute of Fine Chemicals, East China University of Science and Technology Shanghai 200237 China; CSIR-Central Scientific Instruments Organisation Sector-30C Chandigarh 160030 India; Department of Chemistry, University of Texas at Austin Austin Texas 78712-1224 USA sessler@cm.utexas.edu

## Abstract

Precise detection of cellular senescence may allow its role in biological systems to be evaluated more effectively, while supporting studies of therapeutic candidates designed to evade its detrimental effect on physical function. We report here studies of α-l-fucosidase (α-fuc) as a biomarker for cellular senescence and the development of an α-fuc-responsive aggregation induced emission (AIE) probe, termed **QM-NHαfuc** designed to complement more conventional probes based on β-galactosidase (β-gal). Using **QM-NHαfuc**, the onset of replicative-, reactive oxygen species (ROS)-, ultraviolet A (UVA)-, and drug-induced senescence could be probed effectively. **QM-NHαfuc** also proved capable of identifying senescent cells lacking β-gal expression. The non-invasive real-time senescence tracking provided by **QM-NHαfuc** was validated in an *in vivo* senescence model. The results presented in this study lead us to suggest that the **QM-NHαfuc** could emerge as a useful tool for investigating senescence processes in biological systems.

## Abstract 2

Evidence of close association of α-fuc with senescence induction highlights the potential of α-fuc as a novel biomarker for cellular senescence. Here, an α-fuc-responsive AIE probe (**QM-NHαfuc**) allows for the identification of senescent cell *in vivo*.

## Graphical abstract

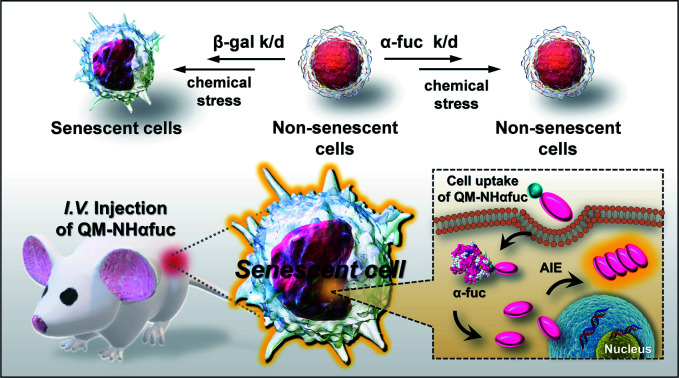

## Introduction

Senescence is a stable state of irreversible cell-cycle arrest necessary for preventing the proliferation of damaged cells,^1–3^ promoting tissue remodeling,^4,5^ and maintaining organism homeostasis.^6,7^ The improper elimination of senescent cells can negatively affect the regenerative capabilities of tissues, thus contributing to local inflammation, age-related degenerative diseases, and cancer stemness and metastasis.^8–12^ In recent decades, studies of cellular senescence have translated into innovative discoveries. For example, the use of genetic ablation or pharmacological senolytic drugs to clear senescent cells can ameliorate and even prevent a wide variety of diseases, such as posttraumatic osteoarthritis.^13–15^ However, there is still a need for convenient tools that can be used to probe the state of senescence and which allow the efficacy of senolytic drugs to be evaluated efficiently.

To identify cellular senescence, numerous biochemical characteristic have been explored, including, for instance, the appearance of condensed nuclear chromatin foci and the upregulation or activation of tumor suppressors (*e.g.*, p53, p21 and p16) that are conducive to cell cycle arrest.^16^ Senescent cells also secrete a wide spectrum of cytokines and growth factors that in principle can be tracked.^17^ Currently, the gold standard for senescence identification is the enzyme lysosomal β-galactosidase, also known as the senescence-associated β-galactosidase (SA-β-Gal).^18–20^ Indeed, SA-β-Gal activity is higher in senescent cells; however, the reliability and specificity of SA-β-Gal as a senescent biomarker has been questioned recently.^21,22^ For instance, in normal human fibroblasts and human cervical cancer cells, the depletion of *GLB1* (a gene encoding lysosomal β-gal) acts to decrease β-gal expression; however, it fails to avert senescence induction.^23^ It follows, therefore, that β-gal is not necessarily a mandatory feature of, or exclusive to, senescence progress. Further complicating matters is that SA-β-Gal activity is also a hallmark of many human cancer types (*e.g.*, colorectal cancer; false-positive) or is only present at low levels in several senescent cell types (*e.g*., epithelial cells; false-negative).^24–31^ As a consequence, reliable identification of senescent cells remains a challenge.

**Fig. 1:**
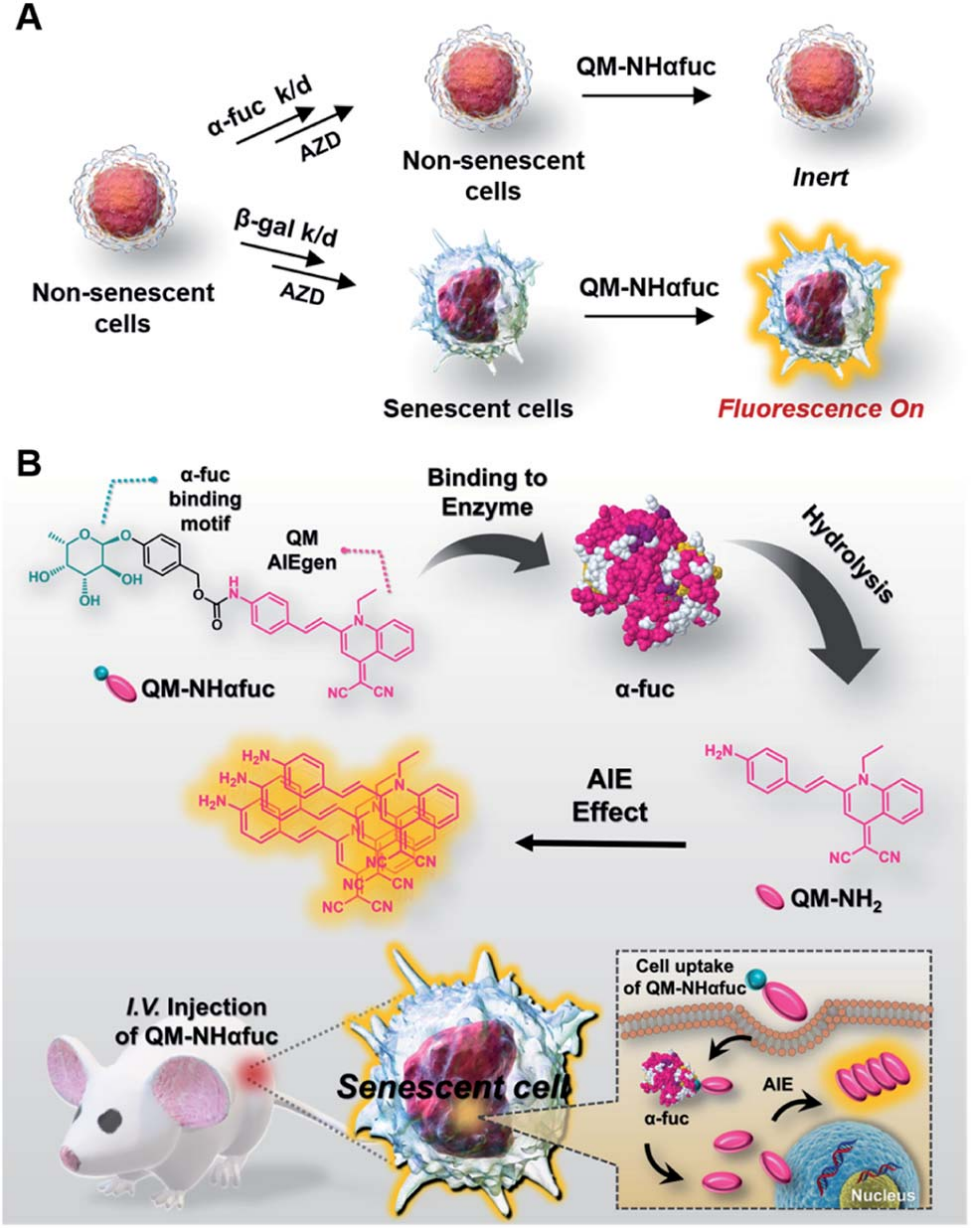
Rational design of a new tool for senescent cells detection. (A) Knockdown of α-fuc but not β-gal prevents the progression of drug-induced senescence; therefore, an α-fuc-responsive probe may be useful in detecting senescent cells lacking β-gal expression. (B) Chemical structure of α-fuc-responsive AIEgen (**QM-NHαfuc**) and schematic illustration of the mechanisms underlying the real-time imaging of senescent cells *in vivo*.

To overcome the perceived limitations associated with SA-β-Gal-based sensors, we have developed and wish to report here a fluorogenic probe that targets α-l-fucosidase (α-fuc) as a senescent biomarker. In humans, there are two types of α-fuc showing different tissue distributions: tissue α-fuc (mainly localized in lysosome) and plasma α-fuc encoded by *FUCA1* and *FUCA2*, respectively.^32^ Recent research provides support for the suggestion that mRNA expression levels and the enzymatic activity of α-fuc encoded by *FUCA1* are upregulated across multiple canonical senescence types, including oncogene-, drug-induced, and replicative cellular senescence.^33^ In the context of the present study, we have confirmed that α-fuc activity is upregulated in diverse senescence models and is correlated with the molecular pathways leading to senescence. As depicted in Fig. 1A, α-fuc knockdown with a small-interfering RNA (siRNA) successfully inhibits the senescence induction triggered by AZD1152-HQPA (AZD; aurora kinase B inhibitor), a cell cycle inhibitor able to elicit cellular senescence.^34–37^ In contrast, pre-treatment of β-gal siRNA under identical conditions does not suppress the senescence progression. Further support for the notion that α-fuc is a useful biomarker for cellular senescence is provided in the present study (*vide infra*). As such, we suggest that selective activity-based fluorogenic probes for α-fuc may have a role to play in achieving the accurate and sensitive detection of senescence, especially in those cases where SA-β-Gal fails to provide precise discrimination of senescent and non-senescent cells. With such considerations in mind, we developed the α-fuc responsive fluorogenic probe (**QM-NHαfuc**) that serves to light up senescent cells through an AIE process *in vitro* and *in vivo* (Fig. 1B).

## Results and discussion

### α-Fuc expression in canonical cellular senescence models

**Fig. 2:**
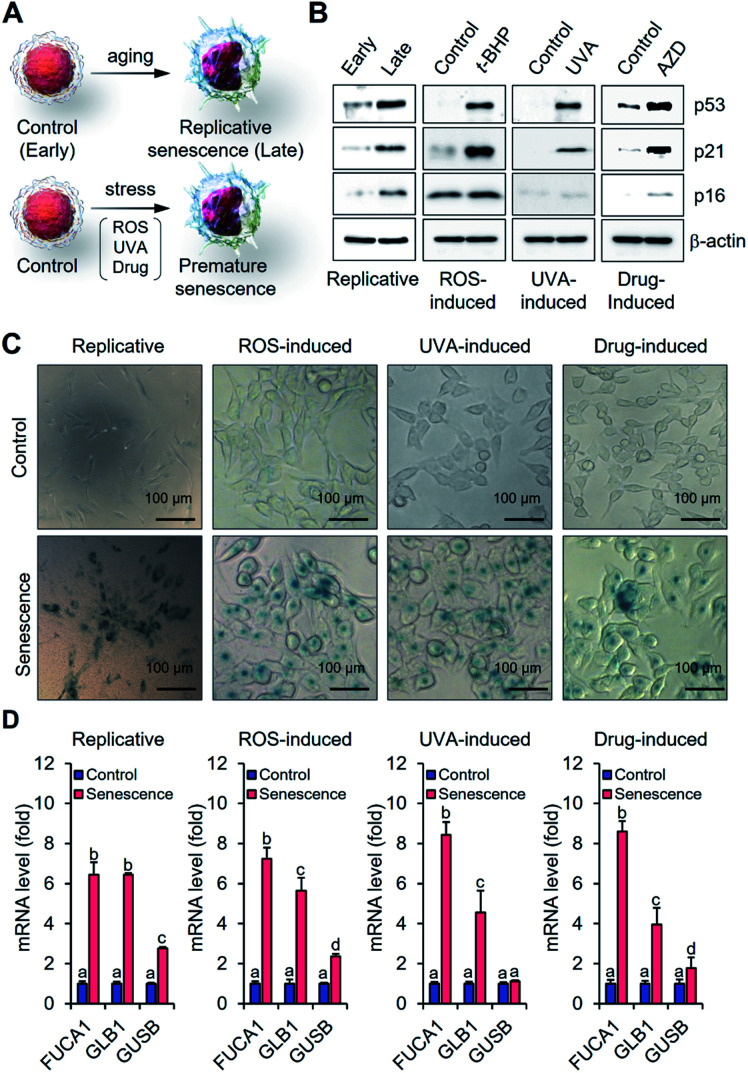
Investigation of α-fuc expression in canonical cellular senescence models. (A) Schematic illustration for generating replicative fibroblast cells and stress-induced premature senescent HCT116 cells. (B) Western blotting of p53, p21 and p16 in early and late fibroblast cells or control and senescent HCT 116 cells. (C) Chromogenic X-fuc assay of early and late fibroblast cells or control and senescent HCT 116 cells. (D) Real-time PCR analysis of *FUCA1*, *GLB1*, and *GUSB* mRNAs in early and late fibroblast cells or control and senescent HCT 116 cells. Data are represented as mean ± SEM (*n* = 3 in D). Statistical significance was determined by a two-way ANOVA test with a *post hoc* Bonferroni test. Different letters (*e.g*., a–d) signify datasets that are statistically distinct (*p* < 0.05).

In an effort to determine whether α-fuc represents a good target for senescence probe development, we tested its expression in different senescence models including replicative and stress-induced premature senescent cells. Human dermal fibroblasts (HDFs) with early passage (p10-11) and late passage (p20-25) were prepared as control and replicative senescent cells, respectively (Fig. 2A). Human colon cancer HCT116 cells were used to generate reactive oxygen species- (ROS-), ultraviolet A- (UVA-), and drug-induced senescence models following treatment with *tert*-butyl hydroperoxide (*t*-BHP; 30 μM for 24 h), UVA (250 mJ cm^−2^, 30 min), or AZD (500 ng mL^−1^ for 3 days), respectively (Fig. 2A). Induction of senescence in each cell was verified by the typical appearance of flattened and enlarged cells and prominent expression of tumor suppressor and cell cycle inhibitors (p53, p21, and p16).^16^ Compared to the control groups, upregulated protein expression and mRNA corresponding to p53, p21, and p16 were observed in each of the senescence models (Fig. 2B and S1†). Blue staining patterns with X-gal indicative of β-gal overexpression and enlarged cell morphology were also observed as expected for the stable induction of senescence (Fig. S2†). Under conditions of a chromogenic X-fuc assay, we exclusively observed an intense blue staining pattern consistent with α-fuc overexpression in all types of senescent cells (Fig. 2C). In addition, real-time polymerase chain reaction analysis revealed elevated *FUCA1* mRNA levels in senescent cells (>6-fold; Fig. 2D). No obvious difference was seen in the case of *FUCA2* (Fig. S3†). On this basis, we rule out artifacts due to possible cross activity of α-fuc encoded by *FUCA2* in the present study. Notably, we found statistically significant lower transcription levels of *GLB1* and *GUSB* (the genes encoding β-gal and β-glucuronidase, respectively) relative to *FUCA1*, leading us to conclude that α-fuc is a more reliable and sensitive marker for senescence identification than β-gal.

### Fidelity assessment of α-fuc as a novel senescence biomarker

**Fig. 3:**
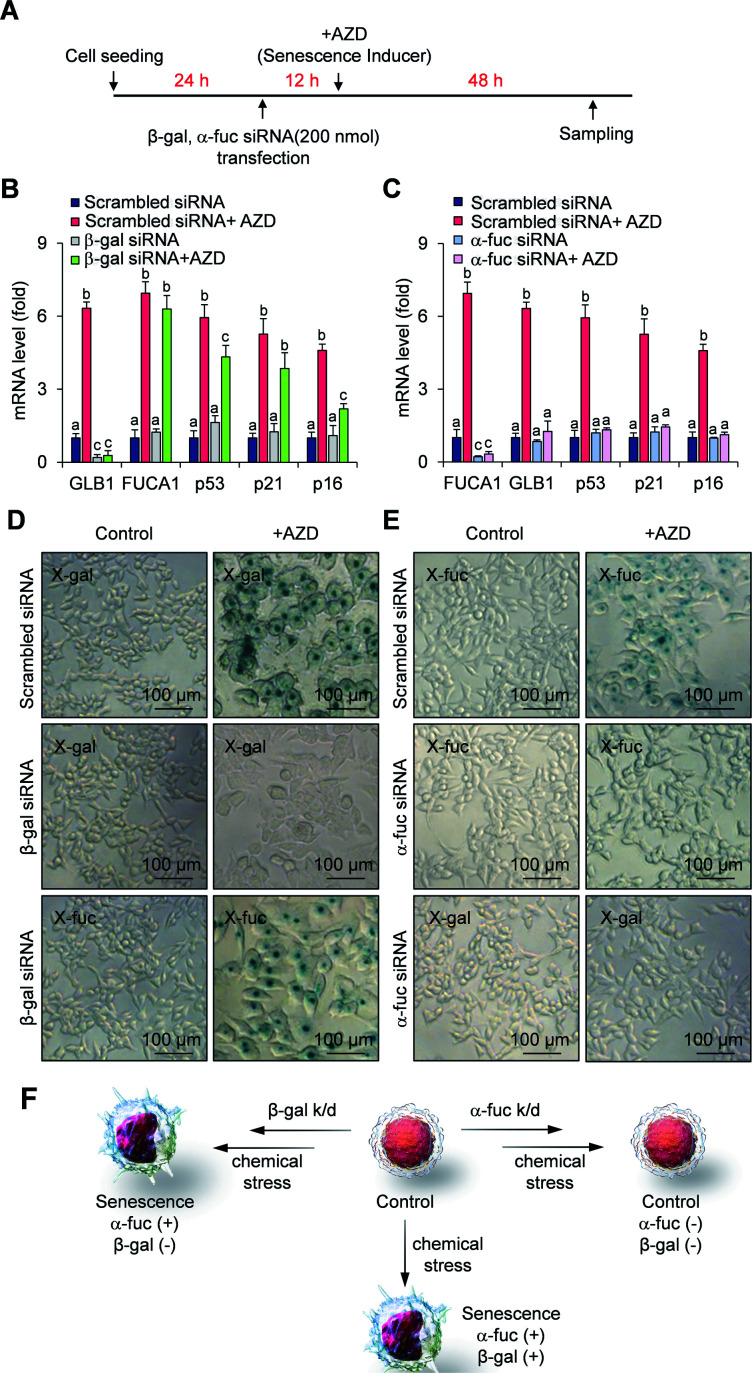
Fidelity assessment of α-fuc as a novel senescence biomarker. (A) Experimental design for the assessment of senescence induction in *GLB1*/*FUCA1*-knockdown cells. (B) mRNA expression of *GLB1*, *FUCA1*, p53, p21, and p16 in *GLB1*-knockdown cells. (C) mRNA expression of *GLB1*, *FUCA1*, p53, p21, and p16 in *FUCA1*-knockdown cells. (D) Chromogenic X-gal and X-fuc assay in *GLB1*-knockdown cells. (E) Chromogenic X-gal and X-fuc assay in *FUCA1*-knockdown cells. (F) Schematic illustration showing correlation of α-fuc and β-gal expression with onset of senescence. Data are represented as mean ± SEM (*n* = 3 in B and C). Statistical significance was determined by a two-way ANOVA test with a *post hoc* Bonferroni test. Different letters (*e.g*., a–c) signify datasets that are statistically distinct (*p* < 0.05).

We next compared the fidelity of β-gal and α-fuc as biomarkers for cellular senescence. The inference that β-gal is not a prerequisite for senescence is based on several observations, including the induction of replicative senescence in fibroblasts from patients with genetic defects in *GLB1* and acquisition of oncogene-induced senescence in *GLB1*-knockdown HeLa cancer cells.^23^ However, to our knowledge no study has examined the potential depletion of the α-fuc encoding gene *FUCA1* during senescence induction. Therefore, we sought to assess the role of α-fuc in maintaining cellular senescence through gene silencing. We found that 200 nmol of β-gal siRNA and α-fuc siRNA were sufficient to decrease by *ca.* 80% the *GLB1* and *FUCA1* mRNA expression levels in senescent HCT116 cells as compared with that in mock-treated cells (Fig. S4†). The effect of *GLB1* and *FUCA1* mRNA expression reduction on generating drug-induced cellular senescence was then evaluated (Fig. 3A). We found that β-gal depletion did not disturb the senescence induction caused by AZD. For instance, HCT116 cells treated with β-gal siRNA as above still showed typical hallmarks of senescence, such as dramatically upregulated p53, p21, p16, and *FUCA1* mRNA expression, as well as enlarged morphologies (Fig. 3B and D). On the other hand, such indicators were statistically less prevalent in HCT116 cells subject to gene *FUCA1* knockdown, implying that α-fuc is required for senescence induction (Fig. 3C and E). An unexpected finding to emerge from the above studies was the recognition that abundant α-fuc was still present in the senescent cells subject to β-gal-depletion and AZD treatment as evidenced from the blue staining pattern seen in a chromogenic X-fuc assay (Fig. 3D). In contrast, no β-gal was observed in otherwise analogous *FUCA1* knockdown cells treated with AZD (Fig. 3E). As summarized in Fig. 3F, expression of α-fuc is more likely to reflect the senescence state in comparison to β-gal. This provides support for the notion that α-fuc is more precise biomarker for senescence progression than the β-gal and that α-fuc responsive fluorogenic probes could serve as a useful tool for detecting senescence even in cells lacking β-gal expression.

### Design, synthesis and characterization of **QM-NHαfuc**

As a test of the suggestion that senescent cells can be tracked *in vitro* and *in vivo* based on α-fuc expression, we sought to create an α-fuc responsive fluorogenic probe. Inspired by the ever-increasing utility of the so-called AIE strategy in developing enzyme activatable probes,^38–40^ we developed an α-fuc responsive AIE luminogen (AIEgen), **QM-NHαfuc**, designed to permit the real-time detection of senescent cells. We selected quinoline–malononitrile (QM) as the AIEgen core and a hydrophilic α-fucopyranoside group as the putative α-fuc recognition unit (Fig. 1B). QM has emerged recently as a useful AIE building block that is endowed with several desirable features, including readily controllable excitation/emission wavelengths, a readily tuneable core structure, excellent photostability, and good biocompatibility.^41–44^

Following the synthetic routes shown in Scheme S1,† the AIEgen **QM-NH2** and the corresponding α-fuc activatable senescence probe **QM-NHαfuc** were prepared (*cf*. Fig. 1B for structures). All new compounds prepared in the course of this study was fully characterized by ^1^H and ^13^C NMR spectroscopy, as well as mass spectrometry (*cf*. ESI†).

**Fig. 4:**
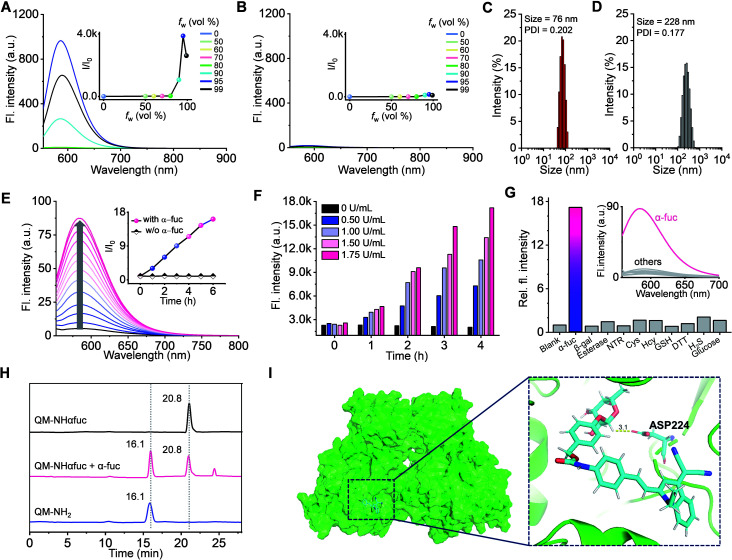
Evaluation of **QM-NHαfuc** as an α-fuc-responsive AIE fluorescence probe. Emission spectra (*λ*_ex_: 543 nm) of (A) **QM-NH2** (10 μM) and (B) **QM-NHαfuc** (10 μM) in a mixture of THF with different aqueous fractions (*f*_w_). Inset: plots of relative fluorescence intensity at 586 nm against *f*_w_. DLS data for (C) **QM-NH2** (5 μM) and (D) **QM-NHαfuc** (5 μM) in pure water. (E) Time-dependent emission spectra (*λ*_ex_: 543 nm) of **QM-NHαfuc** (20 μM) recorded in the presence of α-fuc (2 U mL^−1^). Inset: plots of the relative intensity at 586 nm as a function of time. (F) Fluorescence intensity of **QM-NHαfuc** in the presence of different concentration of α-fuc recorded as a function of time. (G) Fluorescence response of **QM-NHαfuc** (20 μM) after 6 h incubation with α-fuc or other analytes. (H) HPLC monitoring of the hydrolysis of **QM-NHαfuc** seen in the presence of α-fuc. (I) Proposed binding mode for **QM-NHαfuc** within the active site of α-fuc.

The AIE properties of **QM-NHαfuc** and **QM-NH2** were probed using a standard method that involved recording the spectroscopic changes in a tetrahydrofuran (THF)/water-mixed solvent system as a function of the aqueous fraction (*f*_w_).^42–44^ As shown in Fig. S5,† both **QM-NHαfuc** and **QM-NH2** were found to absorb in the 350 to 550 nm spectral range. Increasing the ratio of water in this mixed solvent system led to little change in the absorption spectral features. However, once *f*_w_ exceeded 80%, the fluorescence of **QM-NH2** increased sharply with an emission maximum centered at 586 nm being readily observed (Fig. 4A), as would be expected for an AIE system. In contrast, no such AIE-ascribable response was observed in the case of **QM-NHαfuc**, for which the emission features remained unchanged even when *f*_w_ reached 95% (Fig. 4B).

Both **QM-NH2** and **QM-NHαfuc** were examined by dynamic light scattering (DLS) and scanning electron microscopy (SEM). In the case of **QM-NH2**, tightly packed nano-aggregates were formed in aqueous media (average diameter of 76 nm, uniform; see Fig. 4C and S6†), whereas **QM-NHαfuc** formed looser aggregates (average diameter of 228 nm, non-uniform; see Fig. 4D and S6†). The molecular geometries of **QM-NH2** in the solid-state were then probed. Single-crystal X-ray structural analysis confirmed a *trans* configuration for **QM-NH2** (Fig. S7†). A large dihedral angle (85.1°) is seen for the *N*-ethyl group relative to the QM unit. **QM-NH2** adopts a twisted conformation with a torsional angle of 22.9° between the central ethylene and QM unit, and a 11.0° angle between the central ethylene and phenyl ring. This highly twisted structure presumably serves to preclude close face-to-face stacking, which in turn restricts the extent of aggregation-induced quenching (ACQ) in the solid-state (Fig. S7†). In the case of **QM-NHαfuc**, we propose that (i) the presence of the α-fucopyranoside unit allows for the formation of loosely packed nanostructures with large internal free volumes and (ii) that activation by α-fuc will serve to release **QM-NH2** from **QM-NHαfuc** allowing for formation of tightly packed **QM-NH2** nano-aggregates characterized by an intense AIE fluorescence. This, in turn, should make **QM-NHαfuc** useful for α-fuc-based signal sensing.

### Evaluation of **QM-NHαfuc** as α-fuc-responsive AIE fluorescence probe

Compared to what was seen in the absence of α-fuc (no changes in the spectral features even after 6 h incubation; inset to Fig. 4E and S8†), the fluorescence of the solution containing **QM-NHαfuc** (monitored at 586 nm) was enhanced by *ca.* 16-fold upon incubation with α-fuc (2 U mL^−1^) at 37 °C (Fig. 4E). A positive correlation between the extent of reaction and both time and α-fuc concentration was seen (Fig. 4F). Additionally, an excellent linear relationship (*R* = 0.99) between the fluorescent intensity at 586 nm and the α-fuc concentration was found to hold over the 0–1.75 U mL^−1^ range (Fig. S9†). The detection limit of **QM-NHαfuc** toward α-fuc was calculated to be 1.0 × 10^−2^ U mL^−1^ based on the 3*σ*/slope formula. We thus propose that **QM-NHαfuc** allows for the sensitive detection of α-fuc *in vitro*.

Tests of other potential triggers, revealed that only α-fuc induced an appreciable change in the fluorescence profile under otherwise identical conditions (Fig. 4G). In particular, **QM-NHαfuc** did not show an obvious response to β-gal, a finding that supports our contention that it could provide a useful complement to probes based on SA-β-Gal. Cell culture medium supplemented with different biological entities, such as amino acids, salts, and proteins, did not influence the performance behavior of **QM-NHαfuc** (Fig. S10†). Likewise, the ionic strength of the solution was not found to affect the amphiphilic nature of **QM-NHαfuc** (Fig. S10†).

High-performance liquid chromatography (HPLC) analyses were carried out to confirm the generation of **QM-NH2** from **QM-NHαfuc***in vitro*. As shown in Fig. 4H, a new peak with a retention time of 16.1 min was seen after **QM-NHαfuc** was exposed to α-fuc. This retention time is identical to that of **QM-NH2**, thus supporting the premise that **QM-NH2** is liberated from **QM-NHαfuc** as the result of α-fuc-mediated catalytic hydrolysis. Docking and molecular dynamics (MD) simulations at the atomic level provided support for a thermodynamically favorable interaction between **QM-NHαfuc** and the active site of α-fuc (Fig. 4I). The binding free energy was calculated to be 61.9 kcal mol^−1^. The amino acid residue Asp 224 of the α-fuc domain interacts with C1 of the α-fucopyranoside in **QM-NHαfuc** through hydrogen bonding. Such a finding is consistent with previous binding studies involving α-fucopyranosides.^45,46^ Moreover, **QM-NHαfuc** proved essentially non-toxic in both control and senescent cells even at concentrations up to 100 μM (Fig. S11†). Collectively, these results lead us to suggest that **QM-NHαfuc** could act as an effective α-fuc-specific tool that allows α-fuc activity to be monitored under cellular senescent conditions.

### *In vitro* real-time imaging of senescent cells

**Fig. 5:**
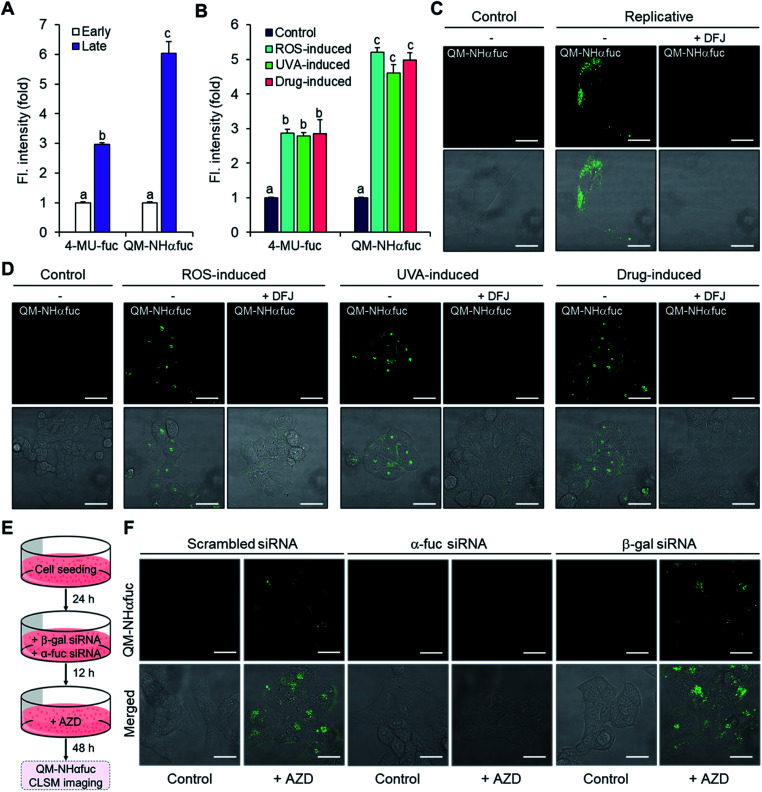
*In vitro* real-time imaging of senescent cells using **QM-NHαfuc**. (A) Change in the fluorescence (senescent *vs.* control) of 4-MU-fuc (30 μM) and **QM-NHαfuc** (30 μM) in replicative senescent cells. (B) Change in the fluorescence (senescent *vs.* control) of 4-MU-fuc (30 μM) and **QM-NHαfuc** (30 μM) in ROS-, UVA-, drug-induced senescent cells. (C) CLSM images of replicative senescent cells using **QM-NHαfuc** (30 μM) with and without pre-treatment of DFJ (100 μM). Scale bar = 50 μm. (D) CLSM images of ROS-, UVA-, drug-induced senescent cells using **QM-NHαfuc** (30 μM) with and without pre-treatment of DFJ (100 μM). Scale bar = 50 μm. (E) Experimental design for the CLSM studies of AZD treated *GLB1*-knockdown and *FUCA1*-knockdown cells using **QM-NHαfuc** (30 μM). (F) CLSM images of AZD treated *GLB1*-knockdown and *FUCA1*-knockdown cells using **QM-NHαfuc** (30 μM). Scale bar = 50 μm. Data are represented as mean ± SEM (*n* = 3 in A and B). Statistical significance was determined by a two-way ANOVA test with a *post hoc* Bonferroni test. Different letters (*e.g*., a–c) signify datasets that are statistically distinct (*p* < 0.05).

Identification of senescent cells on the basis of α-fuc expression was examined using **QM-NHαfuc**. A commercially available α-fuc kit (4-MU-fuc) was used as a positive control. As expected, a distinctive fluorescence increase was observed in each of the senescence types (Fig. 5A and B). It is noteworthy that in our hands **QM-NHαfuc** proved more effective for senescent cell identification than the commercial α-fuc kits. Specifically, while 4-MU-fuc were found to be activated in senescent cells, their response sensitivity proved inferior to that of **QM-NHαfuc**. For example, in replicative senescent cells (late passage, Fig. 5A), **QM-NHαfuc** exhibited 2.0-fold brighter fluorescence than 4-MU-fuc.

While 4-MU-fuc absorbs light in UV spectral region, **QM-NHαfuc** absorbs visible light; this makes it suitable for live senescent cell imaging using confocal laser scanning microscopy (CLSM). As can be seen from an inspection of Fig. 5C, after incubation with **QM-NHαfuc** for 12 h, turn-on fluorescence was observed in replicative senescent cells (4-fold; quantification provided in Fig. S12†). A similar turn on in the fluorescence signal (6-fold increase in intensity) was also noted in ROS-, UVA-, and drug-induced senescent models (Fig. 5D and S12†). However, in non-senescent cells (control group), no change in the fluorescence signature was seen.

Further evidence that the enhanced fluorescence intensity seen in live senescent cells was indeed triggered by α-fuc came from an α-fuc inhibition assay. Here, deoxyfuconojirimycin (DFJ), a commercially available α-fuc inhibitor, was pre-incubated with senescent cells (100 μM for 12 h) prior to **QM-NHαfuc** treatment. Under these conditions, and in contrast to what was seen above, the cellular fluorescence dramatically decreased and even disappeared to all practical extents in the case of the replicative senescence cells (Fig. 5C, D and S12†). A co-localization assay revealed that the fluorescence signal provided by **QM-NHαfuc** in cells is located in the lysosome, suggesting the activation of **QM-NHαfuc** by lysosomal α-fuc (Pearson's *r*: 0.89, Fig. S13†).

We then investigated the fluorescence response of **QM-NHαfuc** in β-gal or α-fuc knockdown models prepared as depicted in Fig. 5E. As described above, β-gal siRNA and AZD treated cells expressed hallmarks of senescence while α-fuc siRNA and AZD treated cells did not (Fig. 3B–E). As shown in Fig. 5F, bright fluorescence was observed in β-gal knockdown senescent cells, a finding that supports our suggestion that **QM-NHαfuc** is a useful tool for tracking senescent cells lacking β-gal expression. Notably, no fluorescence signal was found in α-fuc knockdown cells that did not show any signs of senescence.

### Visualization of senescent cells in mice model

**Fig. 6:**
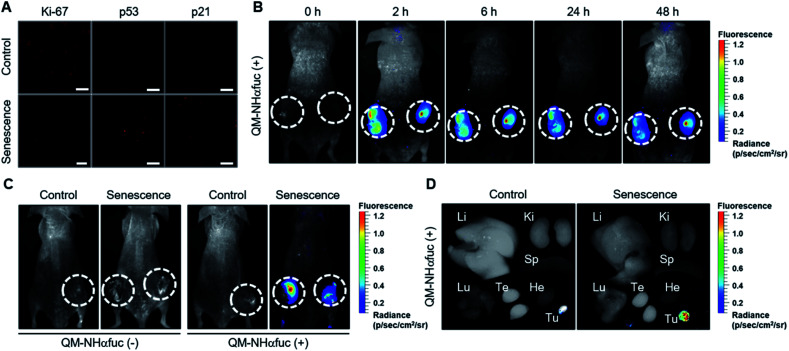
*In vivo* real-time imaging of senescent cells using **QM-NHαfuc**. (A) Immunofluorescence staining for Ki-67, p53, and p21 in control and senescent tumors. Scale bar = 100 μm. (B) *In vivo* fluorescence images of mice bearing senescent tumors recorded at different time points after **QM-NHαfuc** administration. (C) *In vivo* fluorescence images of mice bearing control or senescent tumors, with and without **QM-NHαfuc** administration. (D) *Ex vivo* images of dissected organs from mice bearing control or senescent tumors. Li = liver, Ki = kidney, Sp = spleen, Lu = lung, Te = testis, He = heart, Tu = tumor.

We next sought to explore whether **QM-NHαfuc** might allow for the detection of senescent cells *in vivo*. In accord with a previous report,^47^ HCT116 xenograft mice were administrated AZD for 3 days to generate a drug-induced senescence tumor model. The induction of *in vivo* senescence was confirmed by immunofluorescence staining in tumor tissues, *i.e.*, reduced Ki-67 and increased p53 and p21 expression (Fig. 6A). Once the *in vivo* senescence model was established, **QM-NHαfuc** was injected intravenously and the corresponding *in vivo* fluorescence signal was detected using a Maestro 2 imaging instrument. As shown in Fig. 6B, a rapid fluorescence increase was observed from the lesions treated with AZD. Even 48 h later, bright signals were still maintained, enabling the long-term fluorescence monitoring. Such findings are consistent with the design expectation that **QM-NHαfuc** is a useful probe that allows for the fluorescence-based imaging of senescent cells.

It is important to note that in the absence of the injected probe, the mice treated with AZD displayed no discernible fluorescence. On this basis, we rule out artifacts associated with auto-fluorescence that might arise *inter alia* from senescent cells (Fig. 6C and S14†). Similarly, little fluorescence was observed in the case of control mice that were not subject to AZD treatment whether or not they administered probe **QM-NHαfuc** (Fig. S14†). *Ex vivo* fluorescence imaging of senescent tissues and other organs further revealed that **QM-NHαfuc** only acted to light-up senescent cells (Fig. 6D and S15†). Furthermore, the activities of alanine transaminase (ALT), aspartate transaminase (AST), and serum creatinine, typical markers of hepatic and renal toxicity, remained within the normal range (Fig. S16†).^48^ We thus believe that **QM-NHαfuc** will likely prove safe for use as a senescence imaging probe *in vivo*.

## Conclusions

On the basis of the results presented here, we propose that α-l-fucosidase (α-fuc) is a useful biomarker for cellular senescence and that α-fuc responsive molecular probes could provide a complement to SA-β-Gal probes, which currently account for the majority of the senescence probes currently in use. The upregulation of α-fuc activity was confirmed in diverse senescence models including replicative-, ROS-, UVA-, and drug-induced senescence. Pre-depletion of α-fuc but not β-gal was found to disturb induction of senescence. This is consistent with the close correlation of α-fuc with the molecular pathways of cellular senescence. As an α-fuc responsive molecular probe, **QM-NHαfuc** was successfully developed and found to allow for selective cellular senescence tracing *in vitro* and *in vivo*. The inherently suppressed AIE properties of **QM-NHαfuc** were specifically recovered through enzymatic hydrolysis of the α-l-fucopyranoside unit, allowing for the *in situ* generation of highly fluorescent nanoaggregates. The sensitive, selective, and thermodynamically favored response of **QM-NHαfuc** toward α-fuc was demonstrated through fluorescence emission, HPLC, and molecular docking studies. The readily distinguishable increase in fluorescence emission intensity (4–6-fold increase) seen for **QM-NHαfuc** allowed effective discrimination of replicative-, ROS-, UVA-, and drug-induced senescent cells from control cells *in vitro*. **QM-NHαfuc** could also be used to identify senescent cells lacking β-gal expression. The ability of **QM-NHαfuc** to track senescence was validated in a drug-induced senescence xenograft tumor model. Thus, we suggest that molecular probes or systems that respond to α-fuc could provide a new approach to generating senescent cell-targeting agents or facilitate the screening of potential senolytic drugs.

## Ethical statement

All animal studies were performed in strict accordance with the Korean Animal Protection Act. guidelines (Act. No. 14651, 2017, https://elaw.klri.re.kr/eng_service/lawView.do?hseq=42743&lang=ENG) for the care and use of laboratory animals and was approved by the Korea University Institutional Animal Care and Use Committee (#KUIACUC-2018-57) of Central Laboratory Animal Research Center (Seoul, Korea).

## Data availability

The experimental details and datasets supporting this article are available in the ESI.

## Author contributions

S. K., M. W. and J. S. K conceived the project and designed the experiments. S. K. and W. Y. K carried out the synthetic work. S. K. and C. Y. carried out characterization and solution experiments. M. W. carried out biological experiments and statistical analyses. H. L. carried out docking studies. S. K., M. W., M. L., A. S., Z. G., W.-H. Z., J. L. S, J. Y. L. and J. S. K. wrote and revised the paper. Z. G., W.-H. Z., J. L. S, J. Y. L. and J. S. K. supervised the study. All authors proofread, commented on, and approved the final submitted version of the manuscript.

## Conflicts of interest

The authors declare no competing interest.

## Supplementary Material

SC-012-D1SC02259H-s001

SC-012-D1SC02259H-s002
