# Supplementary material for: Harnessing α-l-fucosidase for in vivo cellular senescence imaging
Source: Chem Sci. 2021 Jun 25;12(29):10054–62. doi: 10.1039/d1sc02259h (PMC8317655; doi:10.1039/d1sc02259h)
Supplement: SC-012-D1SC02259H-s001 [file SC-012-D1SC02259H-s001.pdf]

Electronic Supplementary Material (ESI) for Chemical Science.
This journal is © The Royal Society of Chemistry 2021

Supplemental Information

Harnessing α-L-fucosidase for *in vivo* cellular senescence imaging

Seyoung Koo,[†a] Miae Won,[†a] Hao Li,[†b] Won Young Kim,[a] Mingle Li,[a] Chenxu Yan,[c] Amit Sharma,[d] Zhiqian Guo,[*c] Wei-Hong Zhu,[*c] Jonathan L. Sessler,[*e] Jin Yong Lee,[*b] and Jong Seung Kim[*a]

[a] Department of Chemistry, Korea University, Seoul 02841, Korea.

[b] Department of Chemistry, Sungkyunkwan University, Suwon 16419, Korea.

[c] Institute of Fine Chemicals, East China University of Science and Technology, Shanghai 200237, China.

[d] CSIR-Central Scientific Instruments Organisation, Sector-30C, Chandigarh 160030, India.

[e] Department of Chemistry, University of Texas at Austin, Austin, Texas 78712-1224, United States.

† These authors contributed equally to this work.

Table of Contents

Supplemental Methods	S2-S8
Synthetic Procedures	S9-S11
Supplemental References	S12
Supplemental Figures S1-S16	S13-S22
NMR Spectra, ESI-MS	S23-S31

Supplemental Methods

1. Absorbance and emission spectral changes of QM-NHαfuc and QM-NH_2 in THF/water-mixed solvent system

Each sample was prepared by dissolving 10 mM DMSO stock of **QM-NHαfuc** (10 μM) or **QM-NH_2** (10 μM) in a mixture of tetrahydrofuran (THF) with different aqueous fractions (f_w). As an aqueous medium, pH 7.4 10 mM phosphate buffered saline (PBS) with bovine serum albumin (BSA) was used. Note; concentration of BSA in each sample were adjusted equally to 1 %. Absorbance and emission spectra of each sample was recorded using Jasco V-750 spectrophotometer and Jasco FP-8500 spectrofluorometer, respectively.

2. Fluorescence changes seen for QM-NHαfuc as a function of reaction time and α-fuc concentration

Each sample was prepared by mixing **QM-NHαfuc** (20 μM) and α-L-fucosidase (α-fuc; F884; Sigma Aldrich) (0.05, 0.1, 0.15 and 0.175 U) in 100 μL of an aqueous medium (pH 6.0, 10 mM phosphate buffered saline (PBS) with 1% bovine serum albumin (BSA), 5% tetrahydrofuran (THF); the concentration of BSA and THF in each sample were adjusted equally) and loaded in 96 well plates. After incubation of the samples for the designated time (0, 1, 2, 3 and 4 h) at 37 °C, the fluorescence intensity was recorded using a 575 nm band-pass filter (ex: 535) using a Multi-Detection Microplate Reader system (HIDEX).

3. Selectivity assay

Fluorescence spectra of **QM-NHαfuc** (20 μM) in the presence of α-fuc or other analytes were collected (ex: 543 nm) after incubation of each sample at 37 °C for 6 h. The relative fluorescence intensity at 586 nm of each sample was calculated by dividing each value by the value of the control sample (20 μM **QM-NHαfuc** without any analyte). The nature and concentrations of analytes used for these experiments were as follows: α-fuc (2 U/mL), β-galactosidase (β-gal; 10 U/mL), esterase (10 U/mL), nitroreductase (NTR; 10 U/mL), cysteine (Cys; 2 mM), homocysteine (Hcy; 2 mM), glutathione (GSH; 2 mM), dithiothreitol (DTT; 2 mM), H_2S (2 mM), glucose (2 mM).

4. DLS and SEM measurements

Samples were prepared by dissolving **QM-NHαfuc** (5 μM) in deionized water. The particle size was determined by dynamic light scattering (DLS) using a Malvern Zetasizer Nano S90. Scanning electron microscope (SEM) images were recorded using a JEOL JSM-6360 scanning electron microscope.

5. Single crystal analysis of QM-NH_2

A diffraction grade single-crystal of QM-NH_2 was obtained through the slow evaporation of a DCM/MeOH (v/v = 1:1) solution. Diffraction data were collected with a Bruker D8 Venture X-Ray Diffractometer. The X-

ray crystallographic coordinates for QM-NH_2 have been deposited with the Cambridge Crystallographic Data Centre (CCDC), under deposition number 1996923.

Table S1. Crystal data and structure refinement for QM-NH_2.

Identification code	QM-NH_2
Empirical formula	C22 H18 N4
Formula weight	338.4
Temperature	293(2) K
Wavelength	0.71073 Å
Crystal system	Monoclinic
Space group	P 21/c
Unit cell dimensions	a = 7.3713(4) Å α = 90°.
	b = 11.8588(6) Å β = 92.123(2)°.
	c = 20.5481(14) Å γ = 90°.
Volume	1794.97(18) $Å^3$
Z	4
Density (calculated)	1.252 Mg/m^3
Absorption coefficient	0.076 mm^{-1}
F(000)	712
Crystal size	0.170 x 0.130 x 0.100 mm^3
Theta range for data collection	2.624 to 25.995°.
Index ranges	-9<=h<=9, -14<=k<=13, -25<=l<=16
Reflections collected	8798
Independent reflections	3494 [R(int) = 0.0321]
Completeness to theta = 25.242°	99.20%
Absorption correction	Semi-empirical from equivalents
Max. and min. transmission	0.7456 and 0.6497
Refinement method	Full-matrix least-squares on F^2
Data / restraints / parameters	3494 / 0 / 245
Goodness-of-fit on F2	1.033
Final R indices [I>2sigma(I)]	R1 = 0.0490, wR2 = 0.1109
R indices (all data)	R1 = 0.0868, wR2 = 0.1346
Extinction coefficient	0.027(4)
Largest diff. peak and hole	0.113 and -0.132 e.$Å^{-3}$

6. Reverse-phase HPLC analysis of the reaction of QM-NHαfuc with α-fuc

Aqueous solutions of **QM-NHαfuc** and QM-NH_2 were prepared by mixing each compound (20 μM) in 10 mM PBS (pH 6.0, with 1% BSA) containing 5% THF. The reaction mixture consisting of **QM-NHαfuc** and α-fuc was prepared by mixing **QM-NHαfuc** (20 μM) and α-fuc (2 U/mL) in 10 mM PBS (pH 6.0, with 1% BSA) containing 5% THF. Before analysis, the reaction mixture consisting of **QM-NHαfuc** and α-fuc was incubated at 37 °C for 6 h. Each sample was eluted at a flow rate of 1 mL/min using a mobile phase (Solvent A: Deionized water containing 0.1% TFA, Solvent B: Acetonitrile) consisting of a binary gradient (60% of

Solvent A for 10 min, then 60%-45% of Solvent A for 3 min, then 45% of Solvent A for 5 min, then 45% - 0% of Solvent A for 2 min, then 0% of Solvent A for 5 min, then 0%-60% of Solvent A for 3 min).

7. Docking and molecular dynamic (MD) simulations

The structural data for α-fuc was obtained from the Protein Data Bank (PDB ID: 2ZXD). A grid box was constructed to cover pharmacophores with a spacing of 0.6 Å. Docking parameters were set at 100 runs with 2,500,000 energy evaluations for each cycle using the Autodock 4.2 and AutodockTools 1.5.6 programs and using the Lamarckian genetic algorithm.[1] The 5 structures with the lowest binding energies obtained in this way were used for further molecular dynamics (MD) simulations.

The Gaff force field for **QM-NHαfuc** (ligand) was generated using density functional theory (DFT) calculations carried out employing the M06 exchange function with the 6-31G** basis set by Gaussian 09.[2] The ff14SB force field and Tip3p water models were employed with 5 independent MD simulations being performed using the Amber14 package.[3] The SHAKE algorithm was applied to constrain certain bonds, including those involving hydrogen atoms. The value of the nonbond cutoff was set to 12 Å. The distance between the system and the box edge was more than 10 Å. The entire system was minimized using 5,000 steps via the steepest decent minimization method followed by 5,000 steps employing the conjugate gradient method. The system was then heated from 0 K to 298.15 K within 200 ps with a coupling constant of 2.0 fs for the canonical ensemble (NVT). An isothermal-isobaric ensemble (NPT) using Berendsenbarostat for a constant pressure simulation was employed for 200 ps. Finally, a greater than 2500 ps production run for each MD simulation was performed using a 2.0 fs time step. After evaluating the equilibration process, only the final 2000 ps were used to calculate the binding free energies. The binding energies were obtained using the molecular mechanics generalized-Born surface area (MM/GBSA) method.

8. Cell culture studies

Human colorectal HCT116 cell lines were purchased from the Korean Cell Line Bank (Seoul, Republic of Korea). The cells were cultured in Roswell Park Memorial Institute 1640 (RPMI1640) medium (Hyclone, USA) supplemented with 10% fetal bovine serum (FBS) (GIBCO, USA) and 1% penicillin-streptomycin (Hyclone). Normal human dermal fibroblasts (HDFs) (a kind gift of Professor O. S. Shin; Korea University Guro Hospital) were cultured in RPMI1640 medium supplemented with 15% FBS and 1% penicillin-streptomycin. Cultured cells were maintained at 37 °C in a humidified atmosphere containing 5% CO_2

9. Induction of cellular senescence

Human dermal fibroblasts (HDFs) with early passage (p10-11) and late passage (p20-25) were prepared as control and replicative senescent cells, respectively. HCT116 cells were treated with Aurora kinase B inhibitor (500 ng/mL AZD1152-HQPA for 3 days) as a drug-induced senescence model. Cells used to generate ROS-, UVA-induced senescence models were prepared by treatment with *tert*-butyl hydroperoxide

(30 μM for 24 h), UV-A (250 mJ/cm^2; after 30 min exposure for 24 h), respectively. Cells were analyzed for senescence-induced alterations after the indicated number of incubation days.

10. Small interfering RNA

Small interfering RNA (siRNA) target sequences specific to β-gal and α-fuc were 5′-CUACACAAAUCAGCGAUUU-3′ and 5′-GCAGAGGCGGACA-3′, respectively. The control siRNA sequence used was 5′-CCUACGCCACCAAUUUCGU-3′. The sense and antisense oligonucleotides were purchased from Bioneer (Daejeon, South Korea) and annealed in the presence of an annealing buffer.

11. Cytochemical staining studies of hydrolase activity (X-gal and X-fuc assays)

After fixation in PBS/0.1% glutaraldehyde and washing in PBS, cells were stained at 37 °C for 24 h for hydrolase activities using specific indigogenic substrates in the indicated buffers containing 3 μM potassium ferrocyanide and 3 μM potassium ferricyanide. α-fuc was detected in McIlvain phosphate-citrate buffer, pH 5.0, using 4 mM 5-bromo-4-chloro-3-indolyl-α-L-fucopyranoside (X-fuc, BIOSYNTH). To determine β-galactosidase activities, a Senescence β-Galactosidase Staining Kit (Cell Signaling Technology) was used according to the manufacturer's instructions. Images were obtained using a high-resolution microscope Axioscope (Carl Ziess).

12. Fluorimetric quantification of α-fuc activity.

HCT116 cells were seeded at 3 × 10^3 in 96 well microplates and were allowed to adhere for at least 24 hours. Then, the cells were used to generate ROS-, UVA-, drug-induced senescent cells per the methods described above. For preparing control and replicative senescent cells, HDFs cells with early passage (p10-11) and late passage (p20-25) were seeded at 3 × 10^3 in a 96 well microplate. The cells were allowed to adhere for at least 24 h. Then, 30 μM of **QM-NHαfuc** and 4-MU-fuc in RPMI1640 medium was added to each well. After incubation for 12 h, cells were washed with PBS and the fluorescence intensities were determined using a Multi-Detection Microplate Reader system (HIDEX). The **QM-NHαfuc** fluorescence was collected using a 575 nm band-pass filter with excitation at 535 nm. The fluorescence ascribable to 4-MU-fuc was collected using a 450 nm band-pass filter with excitation at 360 nm.

13. Quantitative real-time PCR

Total RNAs from cell lines were isolated by the PureLink^{TM} total RNA isolation kit (Invitrogen), following the manufacturer's instructions. Reverse transcription to cDNA was performed using the iScript^{TM} cDNA synthesis kit (BioRad, Hercules, CA, USA). All cDNAs used in the real-time PCR studies were normalized with β-actin. Quantitative real-time PCR was carried out using iQ^{TM}SYBR Green Supermix (BioRad). Gene expression was quantified by the delta-delta-CT method, and real-time PCR was performed in a CFX-96 thermal cycler (Applied Biosystems, Foster City, CA, USA). See Table S2 for the primers for each gene.

Table S2. Real-time PCR primers

GENES	5‘ sequences	3‘ sequences
FUCA1	GAAGCCAAGTTCGGGGTGTT	GGGTAGTTGTCGCGCATGA
FUCA2	TTGAGGTAGCCATTAGGAACAGA	GCCGCTTATGGAATGAACTGG
GLB1	TATACTGGCTGGCTAGATCACTG	GGCAAAATTGGTCCCACCTATAA
GUSB	GTC TGC GGC ATT TTG TCG G	CAC ACG ATG GCA TAG GAA TGG
P53	CAGCACATGACGGAGGTTGT	TCATCCAAATACTCCACACGC
21	TGTCCGTCAGAACCCATGC	AAAGTCGAAGTTCCATCGCTC
P16	ATGGAGCCTTCGGCTGACT	GTAACTATTCGGTGCGTTGGG
β-actin	CATGTACGTTGCTATCCAGGC	CTCCTTAATGTCACGCACGAT

14. Western blotting

Protein expression levels of p53, p21 and p16 were determined by western blotting analyses. Briefly, HCT116 cells were seeded at a density of 3 × 10^5 cells in 60 mm dish and incubated for 24 h. Cells were used to generate ROS-, UVA -, drug-induced senescent cells in accord with the method described above. For preparing control and replicative senescent cells, HDF cells with early passage (p10-11) and late passage (p20-25) were seeded at density of 2 × 10^6 in 60 mm dish. The cells were allowed to adhere for at least 24 h. After incubation, the attached cells were washed with ice-cold PBS three times and scraped to create cell pellets. After removal of the PBS, a radioimmunoprecipitation assay (RIPA) lysis buffer containing protease inhibitors provided by the manufacturer (Biosesang) was added into the cell pellets to obtain protein lysates. A Bradford assay was conducted to measure the protein concentration of each cell lysates. Then protein (30 μg/lane) from each cell lysate was loaded onto a sodium dodecyl sulfate poly-acrylamide gel electrophoresis (SDS-PAGE) set up to separate the proteins into individual bands. The separated protein bands were transferred to polyvinylidene difluoride (PVDF) membranes (Merck Millipore) and these membranes were incubated with p53 antibodies (Santa Cruz Biotechnology; SC-126, 1:1000), p21 antibodies (Santa Cruz Biotechnology; SC-397, 1:1000), p16 antibodies (Cell Signaling technology; #92803, 1:1000) and β-actin (Santa Cruz Biotechnology; SC-47778, 1:1000) overnight at 4 °C. The resulting membranes were washed with Tris-buffered saline containing Tween-20 (TBS-T) and then incubated with the anti-rabbit horseradish peroxidase (HRP)-conjugated secondary antibodies (Santa Cruz Biotechnology) and anti-mouse horseradish peroxidase (HRP)-conjugated secondary antibodies (GeneTex) overnight at 4 °C. To detect immunoreactive protein bands, enhanced chemiluminescence reagents (Luminate, Merk Millipore) were used according to the manufacturer’s instructions.

15. *In vitro* cytotoxicity assay

HCT116 and HDF cells were seeded in 96 well plates at 1 × 10^4 cells per well and were allowed to adhere for at least 24 h. Subsequent to incubation, the cells were treated with **QM-NHαfuc** or 1% DMSO as a control for 24 h. To determine the cytotoxicity, a CytoTox96® Non-Radioactive Cytotoxicity Assay Kit (Promega) was used according to the manufacturer's instructions in the presence of the three test agents under study. The absorbance of the wells was detected at 490 nm by a SpectraMax Gemini EM microplate reader (Molecular Devices). Cell viability assays were performed in triplicate and the cytotoxicity was recorded as a percentage calculated for the treated cells relative to the control group.

16. Confocal laser scanning microscope (CLSM) imaging

Senescence induced HCT116 cells (2 × 10^5 cells), control cells (early passage 10) and replicative senescence (late passage 25) HDF cells (2 × 10^5 cells) were seeded on glass bottom dishes and were allowed to adhere for at least 24 h followed by **QM-NHαfuc** (30 μM) treatment for 12 h. For the α-fuc inhibition assay, pre-treatment for 12 h with 100 μM of deoxyfuconojirimycin (DFJ) was made before **QM-NHαfuc** treatment. For the co-localization assay, **QM-NHαfuc** treated cells were washed twice with PBS and the lysosome was stained for 30 min with 500 nM LysoTracker® Red DND-99 (Invitrogen). After incubation, the cells were washed with PBS three times. Fluorescent images were recorded using a confocal laser scanning microscope (Carl Ziess) (excitation: 488 nm, emission wavelengths: 520-600 nm for **QM-NHαfuc** and excitation: 633 nm, emission wavelengths: 650-700 nm for the LysoTracker®).

17. Preparation of senescence mouse model

To establish the *in vivo* senescence model, five-week-old immunodeficient nude mice (nu/nu) (Orient Bio, Inc.) were maintained in pressurized ventilated cages under conditions of repeated controlled illumination (12 h dark; 12 h light) with *ad libitum* access to sterilized water and food (Cat no:1314Fort; Altromin Spezialfutter GmbH & Co.). All animal studies were performed with the approval of the Korea University Institutional Animal Care and Use Committee (#KUIACUC-2018-57) in accord with the Korean Animal Protection Act. HCT116 cells (1 × 10^6) in 50 μL PBS with 50 μL Matrigel® Basement Membrane Matrix (CORNING) were injected subcutaneously into both flanks of each nude mouse. When the tumors had grown to a detectable size (approximately 10 days), the mice were treated with AZD1152-HQPA (30 mg kg^{-1}, day/ single *i.p.* injection; senescence model) or 1 % DMSO (control model) for 3 days.

18. *In vivo* and *ex vivo* diagnostic imaging

When control and senescent xenograft mice were prepared, mice were divided into four sub-groups; 1) control mice treated with 10% DMSO (n = 3), 2) control mice treated with **QM-NHαfuc** (n = 3), 3) senescence mice treated with 10% DMSO (n = 3), 4) senescence mice treated with **QM-NHαfuc** (n = 3). **QM-NHαfuc** samples were prepared by diluting a DMSO stock solution of **QM-NHαfuc** with 10 mM PBS so as to contain a final concentration of 10% DMSO. For the group treated with **QM-NHαfuc**, 8 mg kg^{-1} of **QM-NHαfuc** (100

μL) was administered by means of a single *i.v.* injection. After 1 days, *in vivo* fluorescence images were taken using a Maestro2 instrument (excitation: 488 nm, emission wavelengths: 560-750 nm). To monitor time-dependent fluorescence, images were taken at 0 h, 2 h, 6 h, 24 h and 48 h post-injection of **QM-NHαfuc** in the mice making up group 4 using a Maestro2 instrument (excitation: 488 nm, emission wavelengths: 560-750 nm). To obtain *ex vivo* fluorescence images, animals were euthanized via CO_2 asphyxiation. The xenograft tumors were excised and photographed. *Ex vivo* fluorescence images of the tumors and organs from the control and senescence-induced mice were recorded and quantified using a Maestro2 instrument (excitation: 488 nm, emission wavelengths: 560-750 nm). The fluorescence images and auto-fluorescence were then deconvoluted using the software provided with the above instrument (Maestro software ver.2.4; CRi) using the multi excitation spectral analysis function.

19. Blood toxicity assay

Blood samples of each mouse were centrifuged at 3,000 rpm for 10 min at 4 °C to collect the serum. Then the activities of alanine amino-transferase (ALT), aspartate amino-transferase (AST) and creatinine in the serum were analyzed using the AST and ALT Activity Assay Kits (MAK055, MAK052; Sigma Aldrich) and Creatinine Activity Assay Kits (MAK080; Sigma Aldrich) according to the manufacturer's instructions.

20. Immunohistochemisty

Tissues were extracted and embedded in frozen section compound (Leica Microsystems AG). Tumor sections (5 μm thickness) were produced using a Leica cryotome CM3050S and fixed onto slides and the slides were stored at -70 °C. Immunohistochemistry was performed using anti-Ki-67 (Abcam; ab15580;1: 800), p53 (Santa Cruz Biotechnology; sc-126; 1:100) and p21 (Santa Cruz Biotechnology; sc-397; 1:100) antibodies. Immunohistochemistry was performed according to a IHC standard protocol from Atlas Antibodies company (Atlas Antibodies AB).[4]

Synthetic Procedures

General information and materials

UV-Vis and fluorescence spectra were recorded using a Jasco V-750 spectrophotometer and a Jasco FP-8500 spectrofluorometer, respectively. ESI-MS were obtained using a Shimadzu LC/MS-2020. ^{1}H and ^{13}C NMR spectra were collected on Bruker 500 MHz NMR spectrometers. Reverse-phase HPLC analyses were performed on a Dr. Maish GmbH ReproSil 100 C 18, 5 μm (250 × 4.6 mm) column with a Young Lin HPLC system (YL9100). All chemicals were purchased from Sigma-Aldrich, Alfa Aesar or Tokyo Chemical Industry and used as received. Column chromatography was performed using silica gel 60 (70-230 mesh) as the stationary phase. Analytical thin-layer chromatography was performed using 60 silica gel (pre-coated sheets with 0.25 mm thickness).

Synthesis of QM-NH_2 and QM-NHαfuc

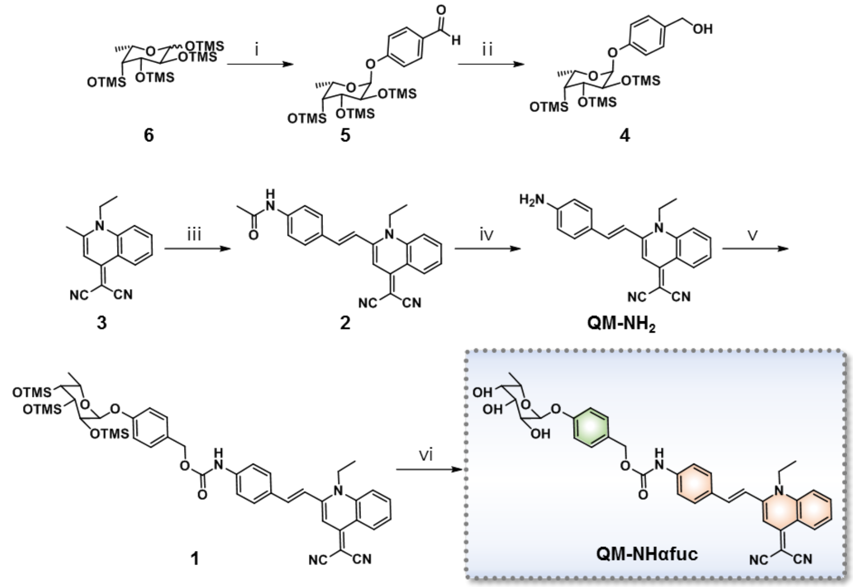

Scheme S1. Synthesis of QM-NH_2 and **QM-NHαfuc**. Reagents and conditions: (i) TMSCl, dichloromethane (DCM), 0 °C, 0.5 h; 4-hydroxybenzaldehyde, TBAI, DIPEA, room temperature (rt), 12 h, 25%; (ii) $NaBH_4$, DCM/MeOH, rt, 2 h, 73%; (iii) 4-actamidobenzaldehyde, piperidine, ACN, reflux, 12 h, 79%; (iv) HCl/dioxane, reflux, 2 h, 32%; (v) phosgene, DIPEA, DCM, 0 °C, 0.5 h; 4, DIPEA, DCM, rt, 6 h, 42%; (vi) Amberlite IR120, DCM/MeOH, rt, 4 h, 81%.

Compounds **6**[5] and **3**[6] were synthesized according to reported procedures.

- **Compound 5**: To a solution of **6**[5] (1.0 g, 2.2 mmol) in anhydrous dichloromethane (DCM, 5 mL) at 0 °C was added TMSI (0.32 mL, 2.2 mmol). The reaction mixture was then stirred for 30 min under argon (Solution A). In a separate round-bottom flask, 4-hydroxybenzaldehyde (0.14 g, 1.1 mmol), TBAI (0.82 g, 2.2 mmol), and molecular sieves (4 Å, 0.2 g) were combined in anhydrous DCM (8 mL) before DIPEA (1.93 mL, 22 mmol) was added (Solution B). Solution B was stirred at rt for 10 min. Then, solution A was

added dropwise into solution B under argon. After the reaction mixture was stirred at rt for 12 h, the volatiles were removed under reduced pressure. The crude product was purified over silica gel using hexanes/EtOAc (v/v, 95:5) as the eluent to yield **5** (0.27 g, 25%): ^{1}H NMR ($CDCl_3$, 500 MHz): δ 9.90 (s, 1H), 7.83 (d, *J* = 8.69 Hz, 2H), 7.19 (d, *J* = 8.69 Hz, 2H), 5.48 (d, *J* = 3.50 Hz, 1H), 4.07 (dd, *J* = 9.62, 3.50 Hz, 1H), 4.01 (dd, *J* = 9.62, 2.63 Hz, 1H), 3.92 (q, *J* = 5.43 Hz, 1H), 3.58 (d, *J* =2.63 Hz, 1H), 1.12 (d, *J* = 5.43 Hz, 3H), 0.19 (s, 9H), 0.17 (s, 9H), 0.07 (s, 9H). ^{13}C NMR ($CDCl_3$, 125 MHz): δ 190.8, 162.6, 131.7, 130.7, 116.8, 98.3, 75.4, 70.9, 68.6, 68.2, 16.6, 0.61, 0.47, 0.19. ESI-MS calc. for $C_{22}H_{40}O_6Si_3Na^+$ [M + Na]$^+$ 507.20; found 507.20.

Compound 4: To a solution of **5** (0.27 g, 0.56 mmol) in DCM/MeOH (v/v, 5:2 mL) at rt was added $NaBH_4$ (0.42 mg, 1.1 mmol). After the reaction mixture was stirred at rt for 2 h, the solvent was removed under reduced pressure. The crude product obtained in this way was purified over silica gel using hexanes/EtOAc (v/v, 3:1) as the eluent to yield **4** (0.20 g, 73%): ^{1}H NMR ($CDCl_3$, 500 MHz): δ 7.34 (d, *J* = 8.74 Hz, 2H), 7.15 (d, *J* = 8.54 Hz, 2H), 5.48 (d, *J* = 2.51 Hz, 1H), 4.55 (d, *J* = 4.12 Hz, 2H), 4.15-4.10 (m, 3H), 3.79 (m, 1H), 2.58 (m, 1H), 1.22 (d, *J* = 5.43 Hz, 3H), 0.30 (s, 9H), 0.27 (s, 9H), 0.19 (s, 9H). ^{13}C NMR ($CDCl_3$, 125 MHz): δ 157.1, 134.4, 128.2, 116.9, 98.6, 75.5, 70.9, 68.7, 67.5, 64.5, 16.5, 0.55, 0.43, 0.14. ESI-MS calc. for $C_{22}H_{42}O_6Si_3Na^+$ [M + Na]$^+$ 509.22, found 509.20.

- **Compound 2**: To a solution of **3**[6] (1.0 g, 4.0 mmol) and 4-acetamidobenzaldehyde (1.4 g, 8.6 mmol) in ACN (15 mL) was added piperidine (0.84 mL, 8.5 mmol). The reaction mixture was then stirred at reflux for 12 h. After the reaction mixture was cooled to rt, the precipitate was filtered and washed with ACN. The crude product obtained in this way was purified by recrystallization to yield the desired product **2** (1.2 g, 79%): ^{1}H NMR (DMSO-d_6, 500 MHz): δ 10.1 (s, 1H), 8.93 (d, *J* = 8.51 Hz, 1H), 8.09 (d, *J* = 8.89 Hz, 1H), 7.93 (m, 1H), 7.77 (d, *J* = 8.65 Hz, 2H), 7.68 (d, *J* =8.65 Hz, 2H), 7.62 (m, 1H), 7.43 (d, *J* = 15.49 Hz, 1H), 7.38 (d, *J* = 15.49 Hz, 1H), 7.02 (s, 1H), 4.57 (q, *J* = 7.15 Hz, 2H), 2.08 (s, 3H), 1.41 (t, *J* = 7.15 Hz, 3H). ^{13}C NMR (DMSO-d_6, 125 MHz): δ 169.1, 152.7, 149.8, 141.3, 139.8, 138.3, 134.2, 130.3, 129.3, 125.6, 125.4, 121.1, 121.0, 119.8, 119.3, 119.2, 118.6, 107.2, 55.3, 47.1, 44.3, 24.5, 14.1. ESI-MS calc. for $C_{24}H_{19}N_4O^-$ [M - H]$^-$ 379.16, found 379.05.
- **QM-NH₂**: A solution of **2** (0.3 g, 0.79 mmol) in dioxane/conc. HCl (v/v, 30: 24 mL) was stirred at reflux for 2 h. After the reaction mixture was cooled to rt, the reaction mixture was neutralized using sat. aqueous Na_2CO_3. When the solution pH reached 7, the precipitate obtained was collected by filtration and washed with water and DCM/MeOH several times. The crude product obtained in this way was purified by recrystallization to yield the desired product **QM-NH₂** (0.085 g, 32%): ^{1}H NMR (DMSO-d_6, 500 MHz): δ 8.91 (d, *J* = 8.47 Hz, 1H), 8.06 (d, *J* = 8.87 Hz, 1H), 7.90 (m, 1H), 7.59 (m, 1H), 7.52 (d, *J* = 8.50 Hz, 2H), 7.30 (d, *J* = 15.49 Hz, 1H), 7.13 (d, *J* = 15.49 Hz, 1H), 7.02 (s, 1H), 6.51 (d, *J* = 8.50 Hz, 2H), 5.81 (s, 2H), 4.55 (q, *J* = 7.11 Hz, 2H), 1.40 (t, *J* = 7.11 Hz, 3H). ^{13}C NMR (DMSO-d_6, 125 MHz): δ 152.1, 151.7, 150.4, 141.5, 138.3, 133.8, 130.5, 125.5, 125.1, 123.0, 121.0, 118.4, 114.0, 113.7, 106.3, 45.9, 44.0, 31.1, 14.1 ESI-MS calc. for $C_{22}H_{17}N_4^-$ [M - H]$^-$ 337.15, found 337.05.

- **Compound 1**: To a solution of **QM-NH_2** (0.085 g, 0.25 mmol), DIPEA (1.0 mL, 23 mmol) in DCM (10 mL) was added phosgene solution (15 wt. % in toluene, 2.30 mL) and stirred at 0 °C for 0.5 h. [Caution: Phosgene is highly toxic; appropriate precautions should be taken to avoid inhalation or exposure.] The excess phosgene was removed by purging with argon for 2 h and was totally removed under reduced pressure. The reaction mixture was then dissolved in DCM (5 mL) to which was added a solution of **4** (0.093 g, 0.19 mmol) and DIPEA (0.066 mL, 0.38 mmol) in DCM (2 mL). After the reaction mixture was stirred at rt for 6 h, the volatiles were removed under reduced pressure. The crude product obtained in this way was purified over silica gel using DCM/MeOH (v/v, 96:4) as the eluent to yield **1** (0.089 g, 42%): ^{1}H NMR ($CDCl_3$, 500 MHz): δ 9.08 (d, *J* = 8.45 Hz, 1H), 7.75 (m, 1H), 7.52 (d, *J* = 8.66 Hz, 1H), 7.52-7.40 (m, 5H), 7.34 (d, *J* =8.63 Hz, 2H), 7.24 (d, *J* =15.70 Hz, 1H), 7.12-7.08 (m, 3H), 6.97 (d, *J* = 15.70 Hz, 1H), 6.90 (s, 1H), 5.39 (d, *J* = 3.05 Hz, 1H), 5.15 (s, 2H), 4.39 (q, *J* = 7.01 Hz, 2H), 4.07-3.97 (m, 3H), 3.68 (d, *J* = 1.40 Hz, 1H), 1.56 (t, *J* = 7.23 Hz, 3H), 1.12 (d, *J* = 6.50 Hz, 3H), 0.19 (s, 9H), 0.17 (s, 9H), 0.08 (s, 9H). ^{13}C NMR ($CDCl_3$, 125 MHz): δ 157.9, 153.3, 153.0, 147.9, 139.6, 138.0, 133.2, 130.0, 129.8, 128.9, 128.6, 128.5, 126.8, 124.5, 121.4, 120.2, 119.2, 118.5, 117.7, 117.0, 116.0, 107.5, 98.5, 75.5, 70.9, 68.7, 67.7, 67.0, 50.9, 43.9, 16.6, 13.9, 0.64, 0.51, 0.24. ESI-MS calc. for $C_{45}H_{57}N_4O_7Si_3^-$ [M - H]$^-$ 849.35, found 849.25.
- **QM-NHαfuc**: A solution of **1** (0.089 g, 0.10 mmol), Amberlite IR 120 (0.1 g) in DCM/MeOH (v/v, 5:1 mL) was stirred at rt for 4 h. The precipitate was filtered and washed with DCM several times to yield **QM-NHαfuc** (0.051 g, 81%): ^{1}H NMR (DMSO-d_6, 500 MHz): δ 10.01 (brs, 1H), 8.93 (d, *J* = 8.42 Hz, 1H), 8.09 (d, *J* = 8.86 Hz, 1H), 7.93 (m, 1H), 7.77 (d, *J* = 8.71 Hz, 2H), 7.62 (m, 1H), 7.56 (d, *J* = 8.42 Hz, 2H), 7.44-7.36 (m, 4H), 7.07 (d, *J* = 8.57 Hz, 2H), 7.02 (s, 1H), 5.41 (d, *J* = 3.10 Hz, 1H), 5.10 (s, 2H), 4.91 (d, *J* = 5.32 Hz, 1H), 4.77 (brs, 1H), 4.62 (d, *J* = 3.99 Hz, 1H), 4.57 (q, *J* = 7.02 Hz, 2H), 3.87 (q, *J* = 6.51 Hz, 1H), 3,76 (m, 2H), 3.56 (brs, 1H), 1.40 (t, *J* = 7.02 Hz, 3H), 1.05 (d, *J* = 6.51 Hz, 3H). ^{13}C NMR (DMSO-d_6, 125 MHz): δ 157.6, 153.7, 152.7, 149.9, 141.2, 139.9, 138.3, 134.1, 130.4, 129.8, 129.7, 129.5, 125.6, 125.4, 121.1, 119.7, 119.0, 118.6, 118.4, 117.0, 107.1, 98.4, 71.8, 70.2, 69.9, 68.0, 67.6, 66.2, 47.1, 44.3, 16.9, 14.1. ESI-MS calc. for $C_{36}H_{33}N_4O_7^-$ [M - H]$^-$ [M-H]$^-$ 633.23, found 633.10.

Supplemental References

1. G. M. Morris, R. Huey, W. Lindstrom, M. F. Sanner, R. K. Belew, D. S. Goodsell and A. J. Olson, *J. Comput. Chem.*, 2009, **30**, 2785-2791.
2. M. J. Frisch, G. W. Trucks, H. B. Schlegel, G. E. Scuseria, M. A. Robb, J. R. Cheeseman, G. Scalmani, V. Barone, G. A. Petersson, H. Nakatsuji, X. Li, M. Caricato, A. Marenich, J. Bloino, B. G. Janesko, R. Gomperts, B. Mennucci, H. P. Hratchian, J. V. Ortiz, A. F. Izmaylov, J. L. Sonnenberg, D. Williams-Young, F. Ding, F. Lipparini, F. Egidi, J. Goings, B. Peng, A. Petrone, T. Henderson, D. Ranasinghe, V. G. Zakrzewski, J. Gao, N. Rega, G. Zheng, W. Liang, M. Hada, M. Ehara, K. Toyota, R. Fukuda, J. Hasegawa, M. Ishida, T. Nakajima, Y. Honda, O. Kitao, H. Nakai, T. Vreven, K. Throssell, J. A. Montgomery, Jr., J. E. Peralta, F. Ogliaro, M. Bearpark, J. J. Heyd, E. Brothers, K. N. Kudin, V. N. Staroverov, T. Keith, R. Kobayashi, J. Normand, K. Raghavachari, A. Rendell, J. C. Burant, S. S. Iyengar, J. Tomasi, M. Cossi, J. M. Millam, M. Klene, C. Adamo, R. Cammi, J. W. Ochterski, R. L. Martin, K. Morokuma, O. Farkas, J. B. Foresman, and D. J. Fox, Gaussian, *Gaussian 09, Revision D.01*, Inc., Wallingford CT, 2016.
3. D. A. Case, V. Babin, J. T. Berryman, R. M. Betz, Q. Cai, D. S. Cerutti, T. E. Cheatham, III, T. A. Darden, R. E. Duke, H. Gohlke, A. W. Goetz, S. Gusarov, N. Homeyer, P. Janowski, J. Kaus, I. Kolossváry, A. Kovalenko, T. S. Lee, S. LeGrand, T. Luchko, R. Luo, B. Madej, K. M. Merz, F. Paesani, D. R. Roe, A. Roitberg, C. Sagui, R. Salomon-Ferrer, G. Seabra, C. L. Simmerling, W. Smith, J. Swails, R. C. Walker, J. Wang, R. M. Wolf, X. Wu and P. A. Kollman, *AMBER 14*, University of California, San Francisco, 2014.
4. The "IHC Standard Protocol", can be found under https://www.atlasantibodies.com/resources/antibody-applications/protocols/ihc-standard-protocol/.
5. T. Uchiyama and O. Hindsgaul, *Synlett*, 1996, **6**, 499-501.
6. C. X. Shi, Z. Q. Guo, Y. L. Yan, S. Q. Zhu, Y. S. Xie, Y. S. Zhao, W. H. Zhu and H. Tian, *ACS Appl. Mater. Inter.*, 2013, **5**, 192-198.

Supplemental Figures

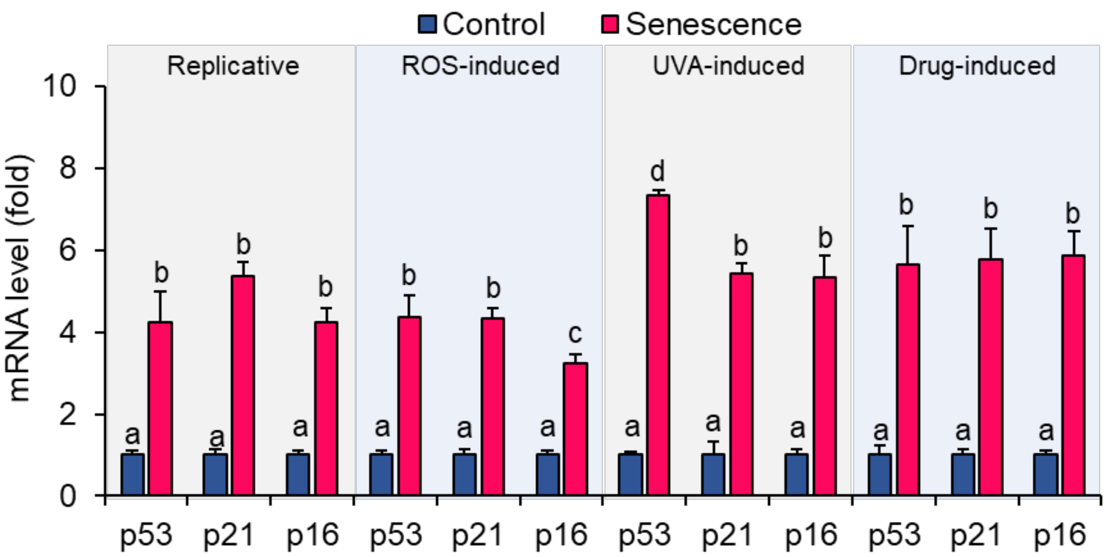

Fig. S1 Real-time PCR analysis of p53, p21, and p16 mRNAs in control and various senescent-induced cells. Data are represented as mean ±SEM (n = 3). Statistical significance was determined by a one-way/two-way ANOVA test with a post-hoc Bonferroni test. Different letters (*e.g.*, a–d) signify datasets that are statistically distinct ($p < 0.05$).

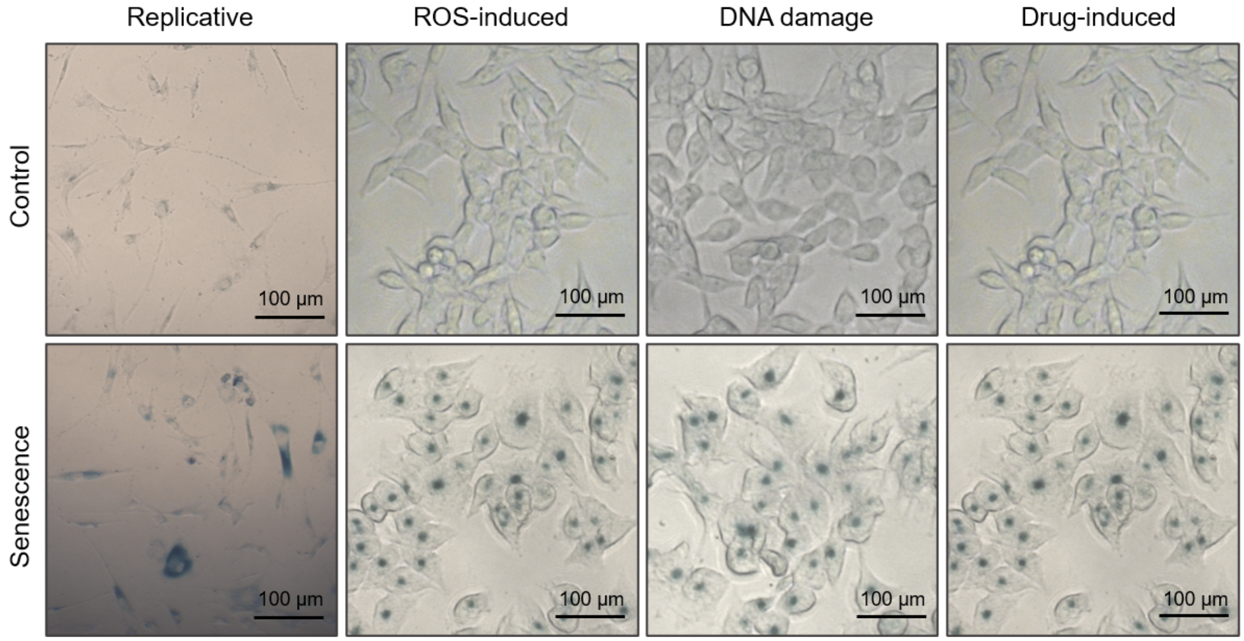

Fig. S2 Chromogenic X-gal assay of control and various senescent-induced cells.

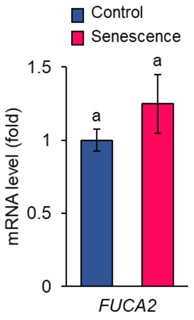

Fig. S3 Real-time PCR analysis of human *FUCA2* in control and drug-induced senescent HCT116 cells. Data are represented as mean ±SEM (n = 3). Statistical significance was determined by a one-way ANOVA test with a post-hoc Bonferroni analysis. The use of the same letter (*e.g.*, a) signify data sets with no statistical difference for a p < 0.05 cutoff.

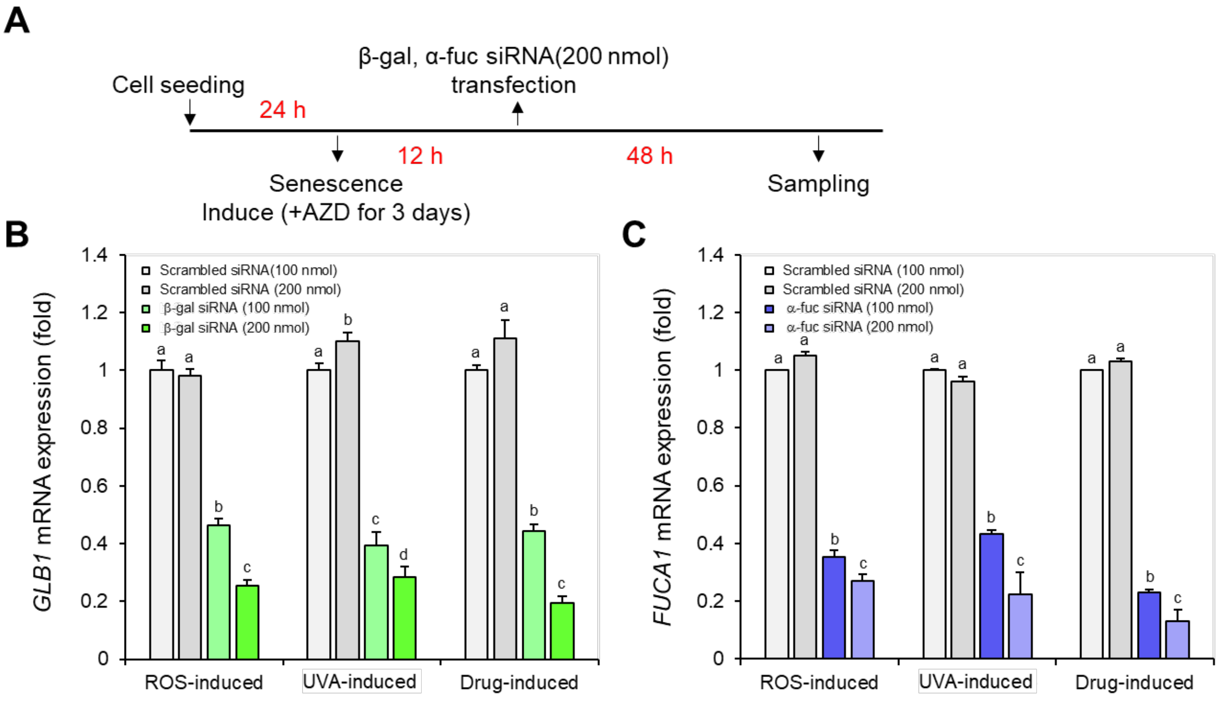

Fig. S4 β-gal, α-fuc siRNA transfection in senescent cells. (A) Experimental design for the investigating *GLB1*/*FUCA1*-knockdown efficiency in senescent cells. (B) Relative *GLB1* mRNA expression level in ROS-, UVA-, and drug-induced senescent cells after transfecting with scrambled siRNA or β-gal siRNA. (C) Relative *FUCA1* mRNA expression level in ROS-, UVA-, and drug-induced senescent cells after transfecting with scrambled siRNA or α-fuc siRNA. Data are represented as mean ±SEM (n = 3). Statistical significance

was determined by a one-way ANOVA test with a post-hoc Bonferroni test. Different letters (*e.g.*, a–d) signify datasets that are statistically distinct ($p < 0.05$).

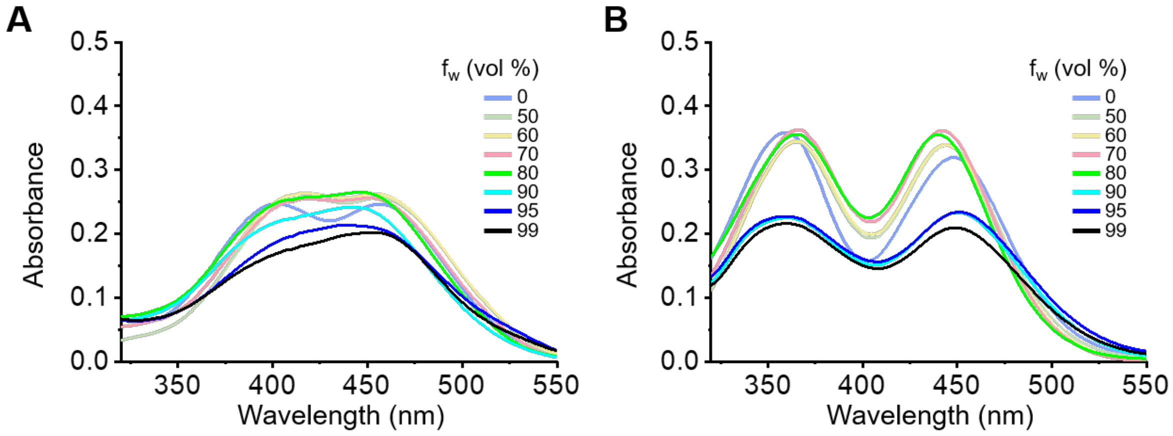

Fig. S5 Absorption spectra of QM-NH_2 and **QM-NHαfuc**. (A) Absorption spectra of QM-NH_2 (10 μM) in a mixture of THF with different fractions (f_w) of an aqueous medium (pH 7.4, 10 mM PBS with 1% BSA). (B) Absorption spectra of **QM-NHαfuc** (10 μM) in a mixture of THF with different fractions (f_w) of an aqueous medium (pH 7.4, 10 mM PBS with 1% BSA).

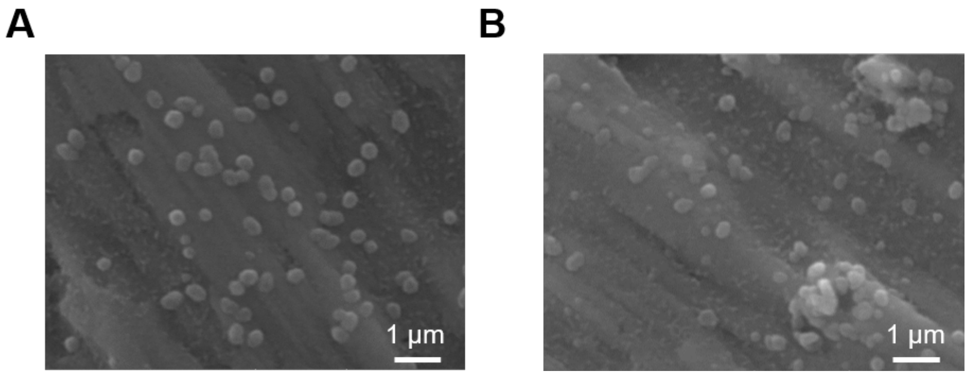

Fig. S6 SEM analysis. (A) SEM data for QM-NH_2 (5 μM) in pure water. (B) SEM data for **QM-NHαfuc** (5 μM) in pure water.

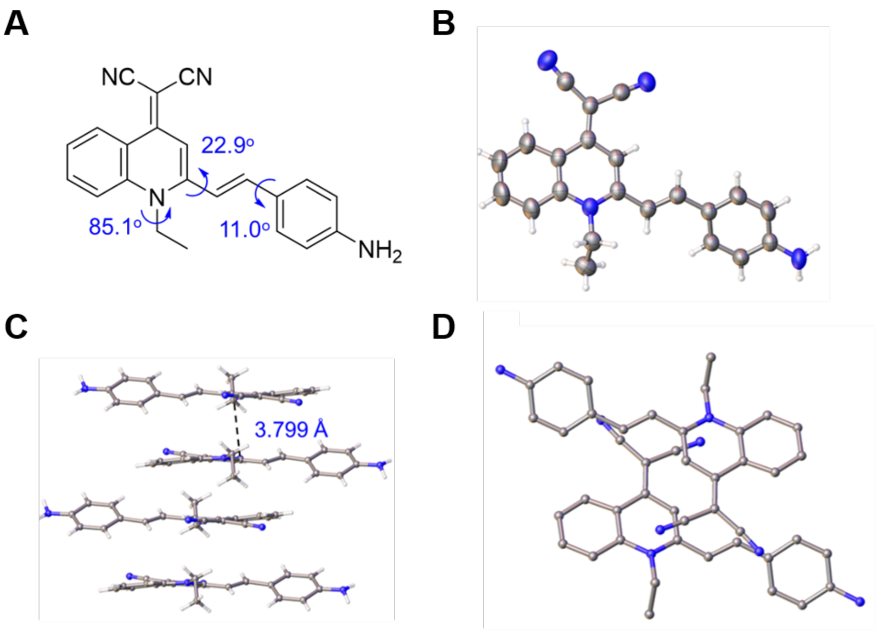

Fig. S7 Single crystal X-ray diffraction analysis of QM-NH_2. (A) Values of key dihedral angles meant to illustrate the twisted structure of QM-NH_2 seen in the solid state as deduced from a single crystal X-ray diffraction analysis. (B) Crystal structure of QM-NH_2. (C-D) Molecular stacking arrangement of QM-NH_2

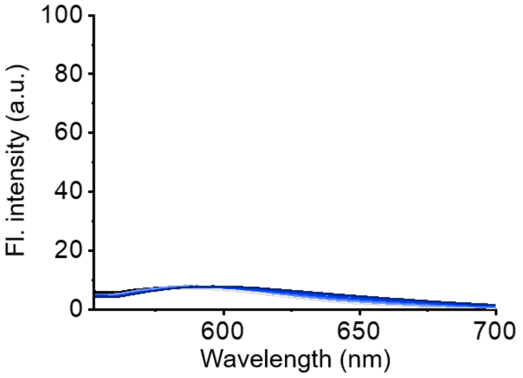

Fig. S8 Time-dependent emission spectra (recorded over the course of 6 h) of **QM-NHαfuc** (20 μM) without α-fuc in an aqueous medium (pH 6.0, 10 mM PBS with 1% BSA, 5% THF).

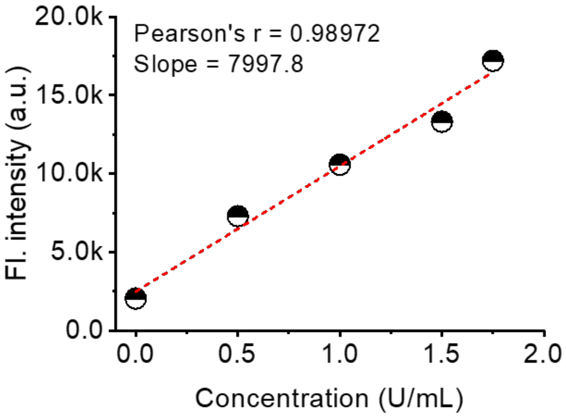

Fig. S9 Linear relationship between the fluorescence intensity of **QM-NHαfuc** (20 μM) and the α-fuc level. Note: The detection limit was calculated to be 1.0 × 10^{-2} U/mL (3σ/slope).

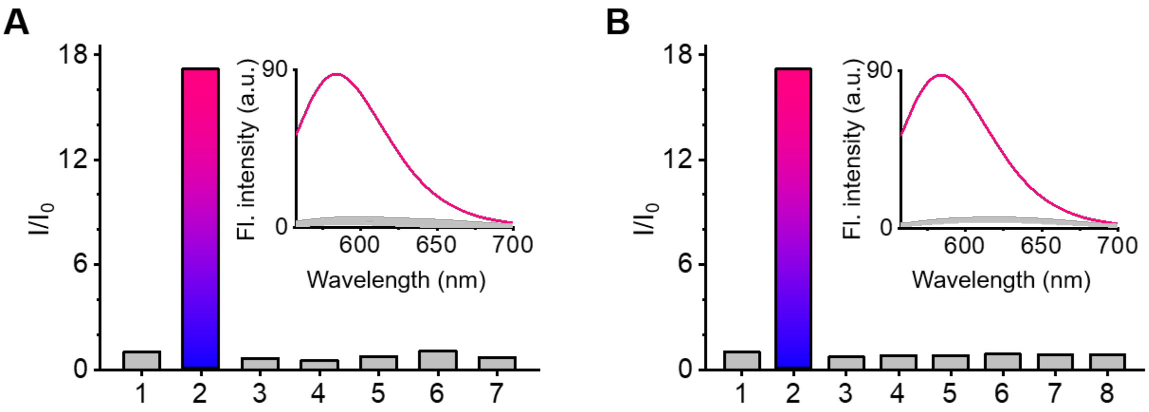

Fig. S10 Selectivity assay. (A) Fluorescence response of **QM-NHαfuc** (20 μM) in the presence of varying concentrations of aqueous NaCl.; 1: Blank, 2: α-fuc (2 U/mL), 3: 0 mM NaCl, 4: 200 mM NaCl, 5: 400 mM NaCl, 6: 600 mM NaCl, 7: 800 mM NaCl, 8: 1000 mM. (B) Fluorescence response of **QM-NHαfuc** (20 μM) in cell culture medium.; 1: Blank, 2: α-fuc (2 U/mL), 3: Minimum Essential Medium Eagle (MEM) (with 10% FBS), 4: Dulbecco's Modified Eagle's Medium (DMEM) (with 10% FBS), 5: Dulbecco's Modified Eagle's Medium /F-12 Nutrient Mixture Ham's (DMEM/F12) (with 10% FBS), 6: RPMI 1640 (with 10 % FBS), 7: McCoy's 5A (with 10% FBS). In case of the samples containing α-fuc, the solution was prepared in an aqueous medium (pH 6.0, 10 mM PBS with 1% BSA, 5% THF). I/I_0: fluorescence intensity ratio at 586 nm of each sample, I_0: fluorescence intensity of the blank sample. All fluorescence spectra were recorded after 6 h incubation at 37 °C.

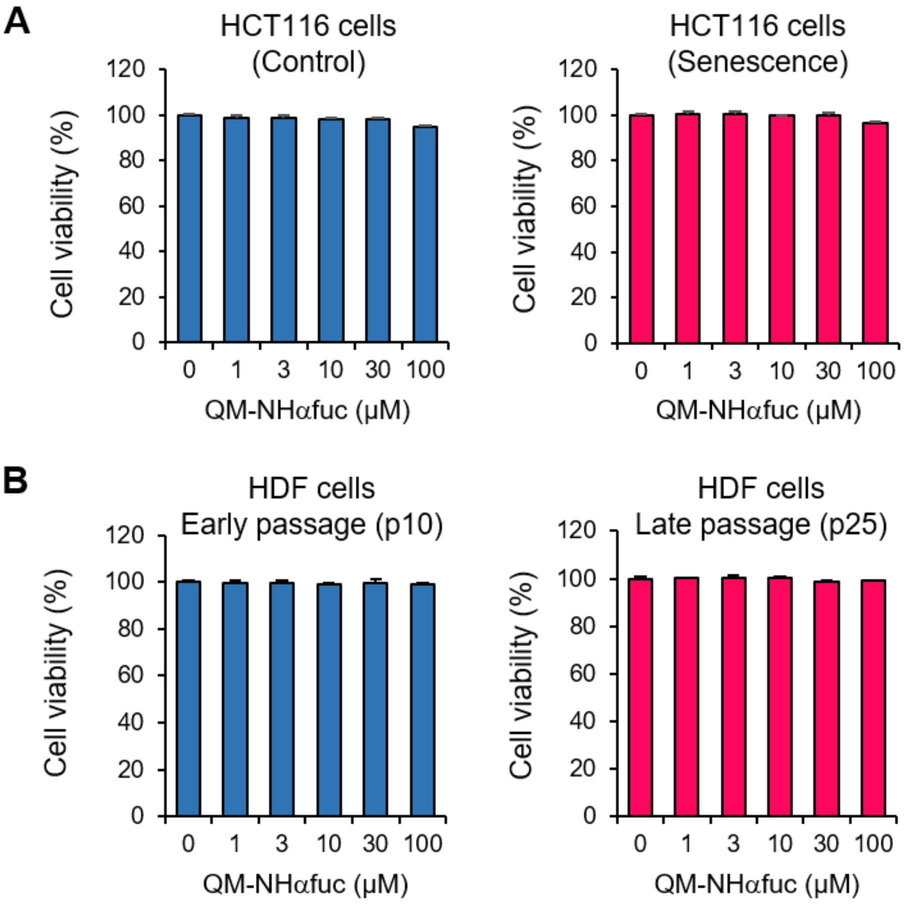

Fig. S11 Cell viability assay. (A) Cell viability of control and senescence (AZD1152-HQPA treated) HCT116 cells treated with **QM-NHαfuc**. (B) Cell viability of early passage (p10) and late passage (p25) HDF cells treated with **QM-NHαfuc**. Control and senescent cells were treated with different concentration of **QM-NHαfuc**, or 1% DMSO (control) for 24 h. CytoTox96® Non-Radioactive Cytotoxicity Assays were used to measure cell viability.

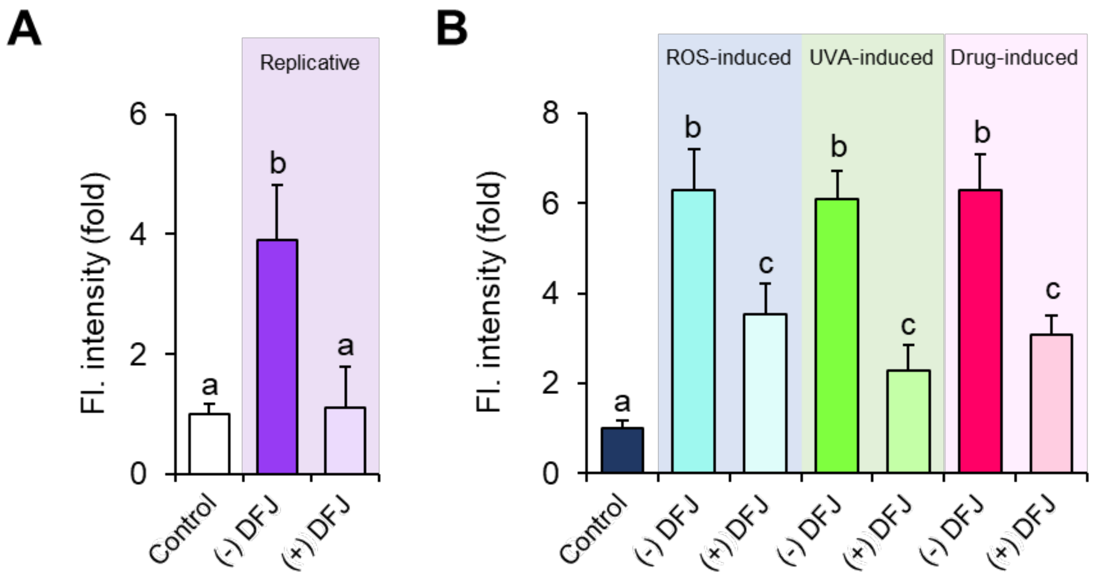

Fig. S12 Quantified fluorescence intensity of CLSM images using ImageJ. (A) Fluorescence intensity of **QM-NHαfuc** with and without DFJ treatment in early passage (control) and late passage (replicative) cells. (B) Fluorescence intensity of **QM-NHαfuc** with and without DFJ treatment in control and senescent (ROS-, UVA-, and drug-induced) cells. Data are represented as mean ±SEM. Statistical significance was determined by a two-way ANOVA test with a post-hoc Bonferroni test. Different letters (*e.g.*, a–c) signify data sets that are statistically distinct ($p < 0.05$).

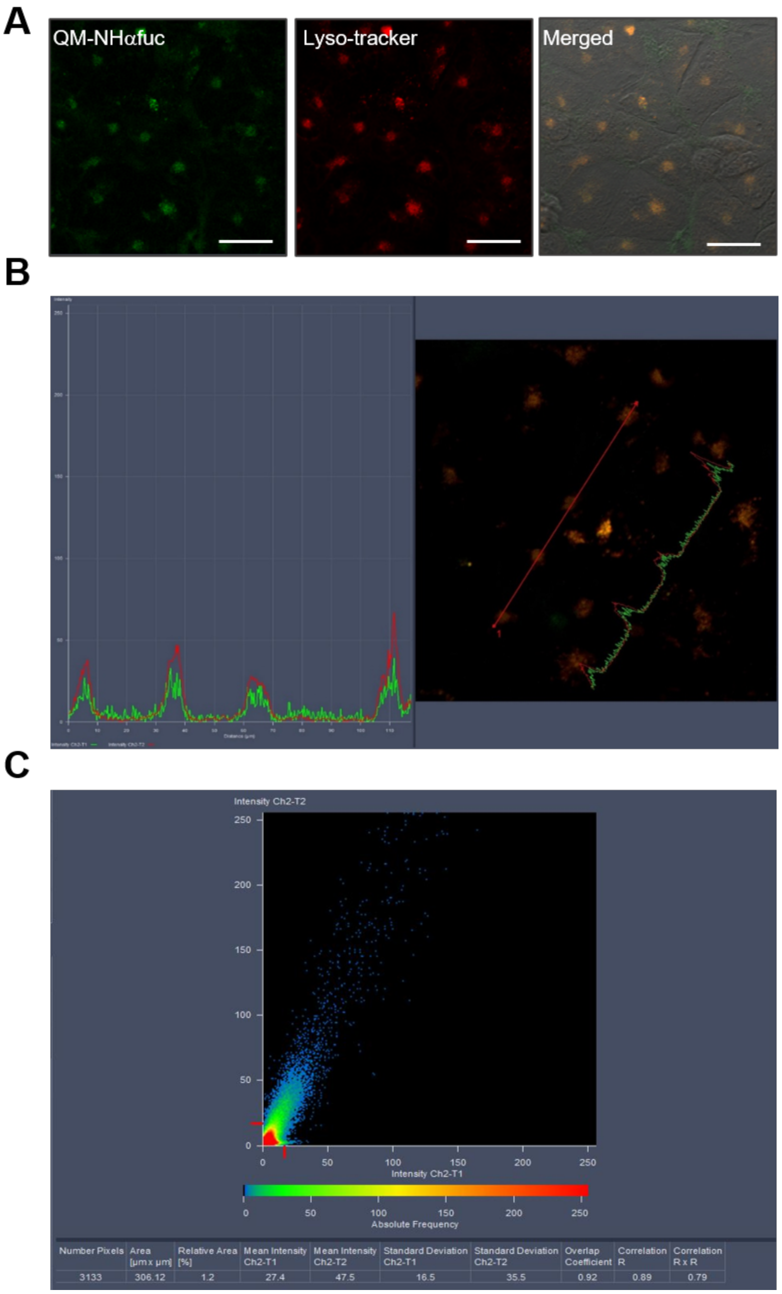

Fig. S13 Subcellular co-localization assay. (A) Subcellular co-localization of the fluorescence signal from **QM-NHαfuc** (30 μM) and LysoTracker® Red DND-99 (500 nM). Scale bar = 50 μm. (B) Scatter plot. (C) Co-localization histogram of the fluorescence signal from **QM-NHαfuc** and LysoTracker® Red DND-99.

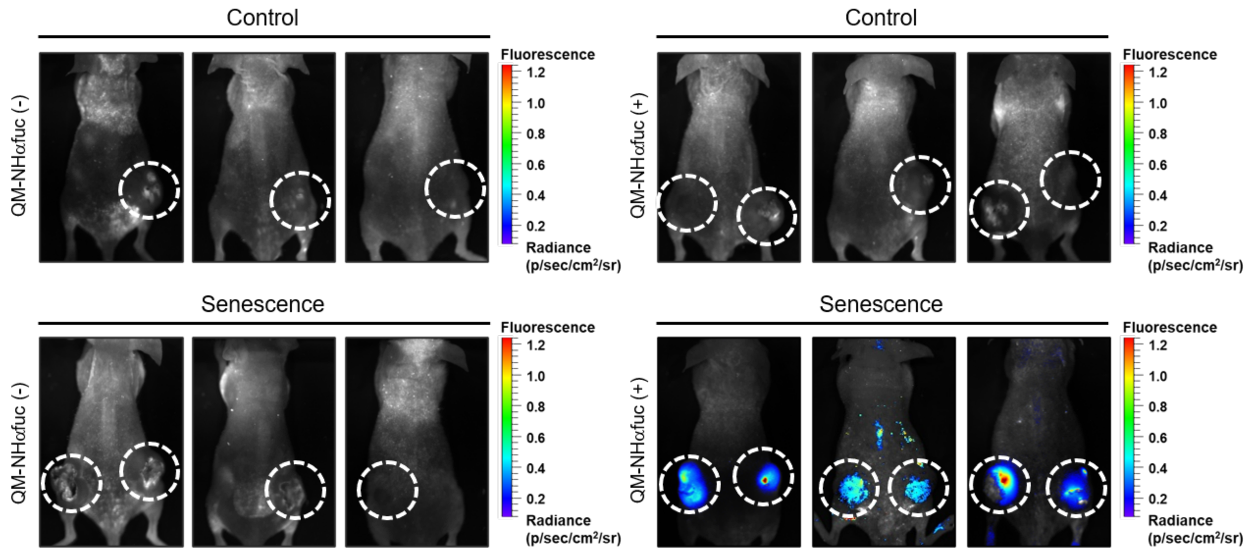

Fig. S14 *In vivo* fluorescence images of mice bearing control and senescent-induced tumors (AZD1152-HQPA treated). Images were taken with and without administration of **QM-NHαfuc** (n = 3/group).

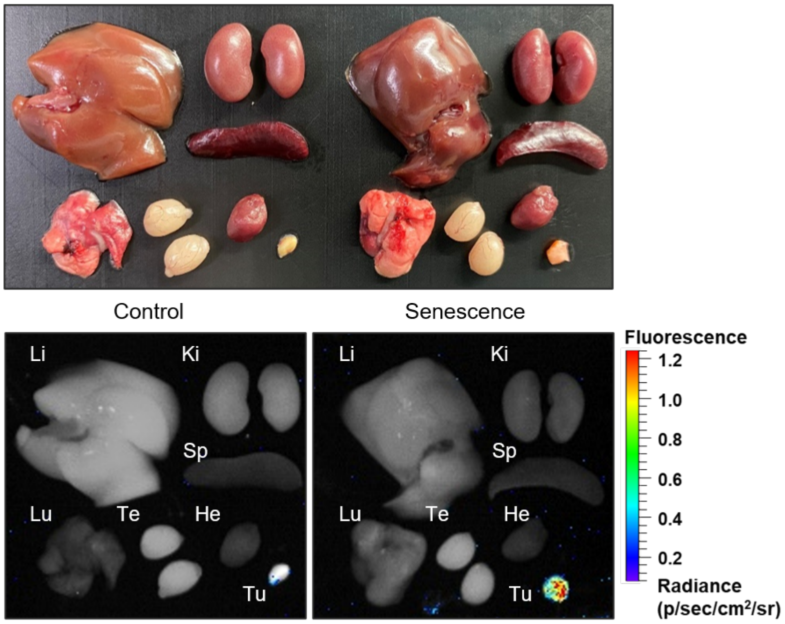

Fig. 15 *Ex vivo* images of dissected organs from mice bearing control tumors and senescent-induced tumors. Li = liver, Ki = kidney, Sp = spleen, Lu = lung, Te = testis, He = heart, Tu = tumor.

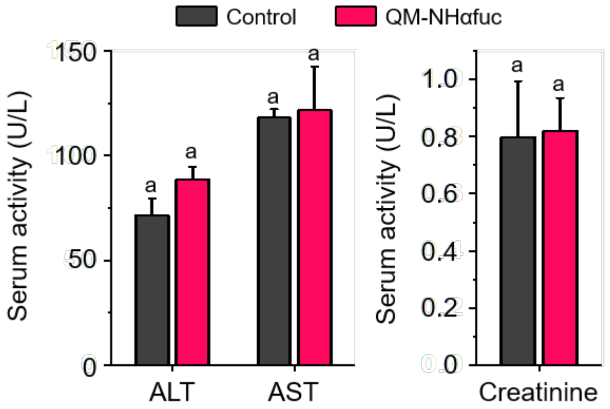

Fig. S16 Hepatic and renal toxicity assays in mice treated with 10% DMSO (control) and **QM-NHαfuc**. Data are represented as mean ±SEM (n = 3). Statistical significance was determined by a one-way ANOVA test with a post-hoc Bonferroni test. The use of the same letter (*e.g.*, a) signify data sets with no statistical difference ($p < 0.05$).

NMR Spectra, ESI-MS

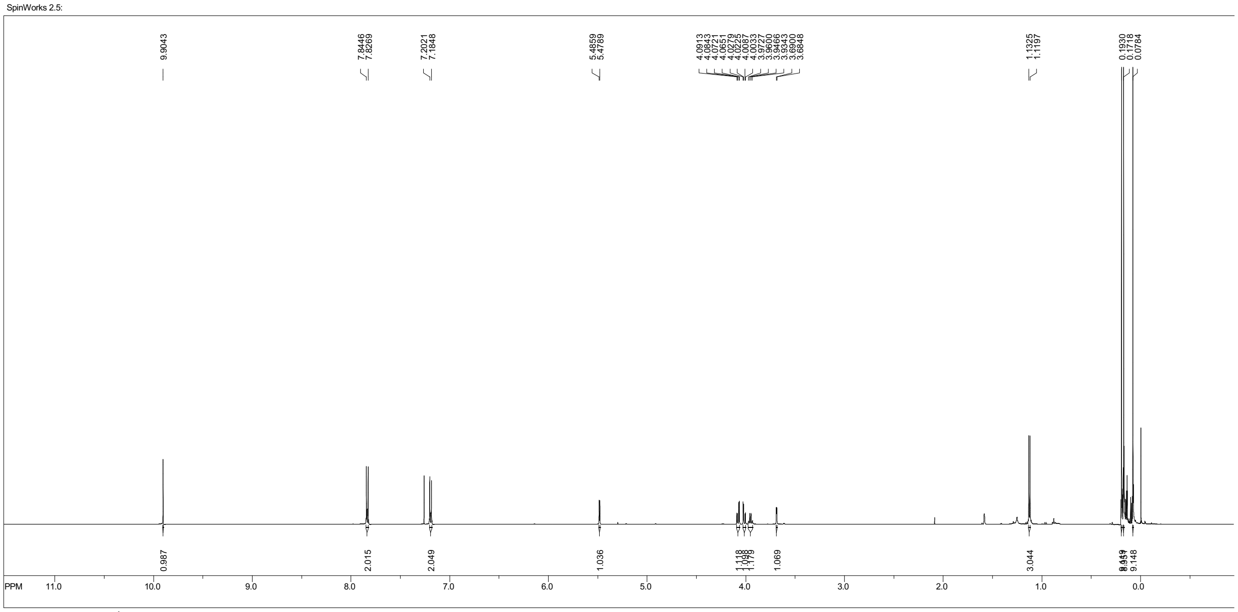

Fig. S16 ^{1}H NMR spectrum (500 MHz) of **5** in $CDCl_3$.

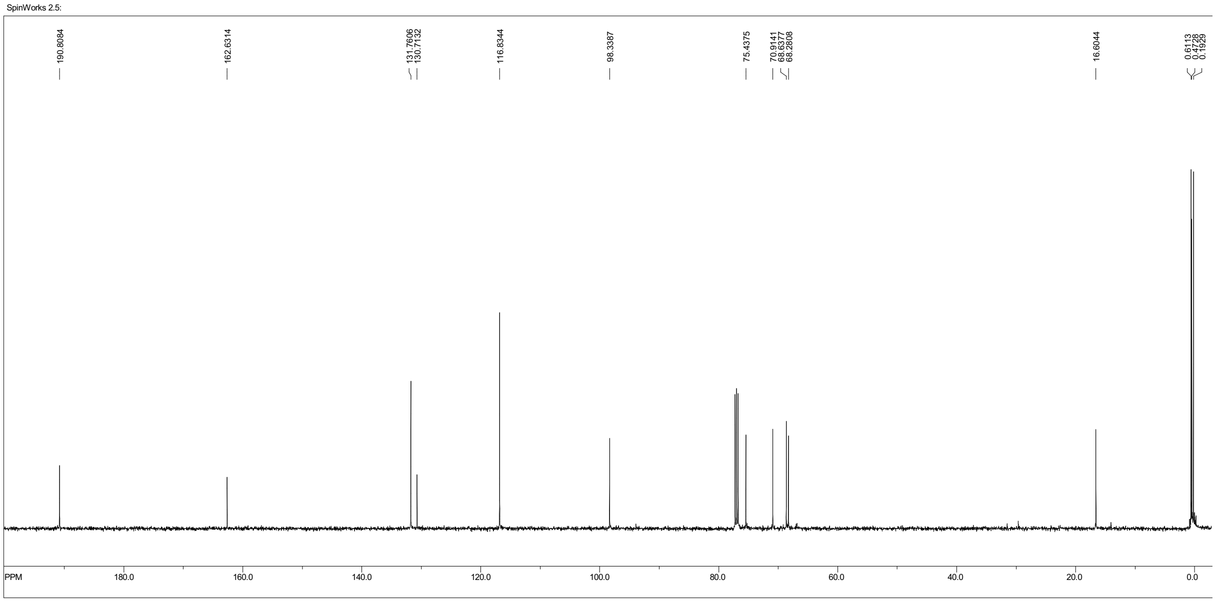

Fig. S17 ^{13}C NMR spectrum (125 MHz) of **5** in $CDCl_3$.

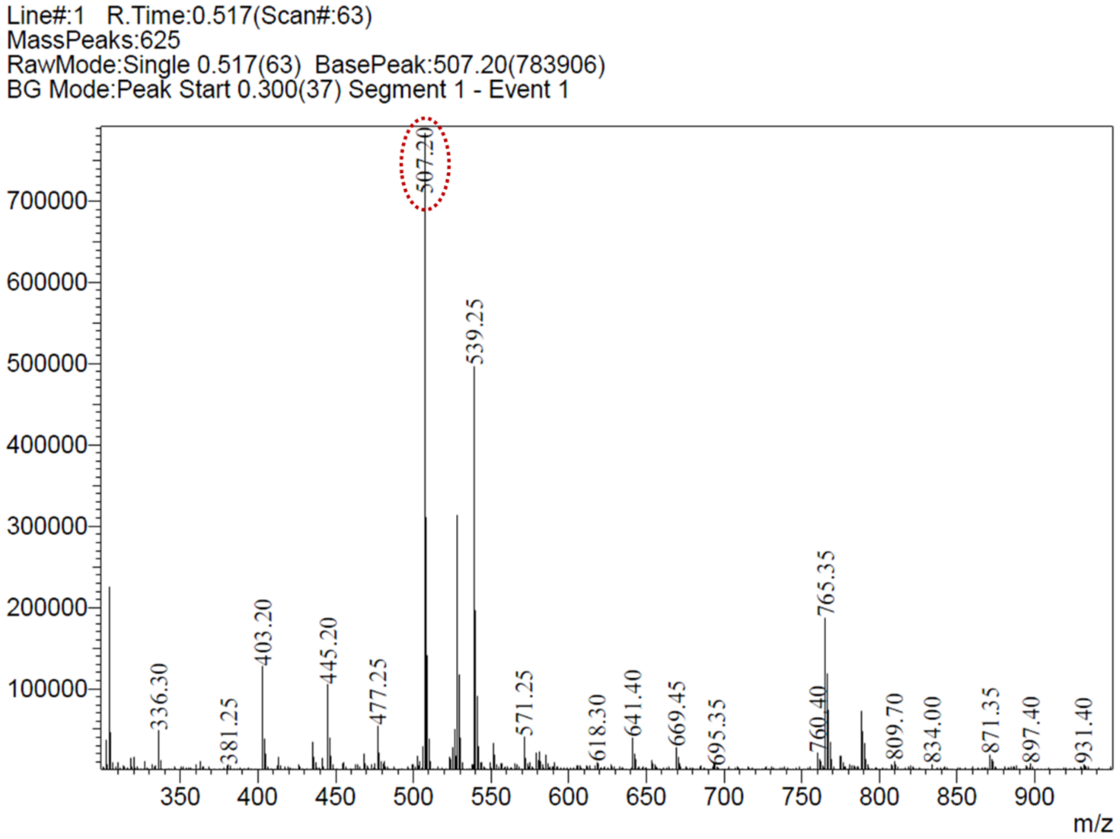

Fig. S18 ESI-MS spectrum of **5**.

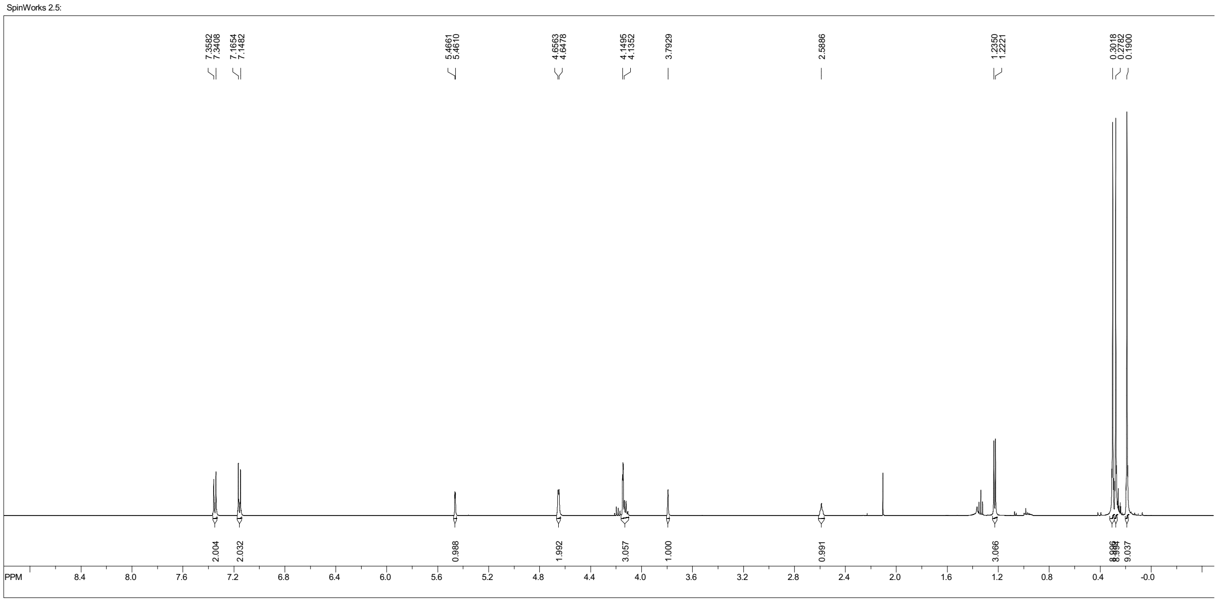

Fig. S19 ^{1}H NMR spectrum (500 MHz) of **4** in $CDCl_3$.

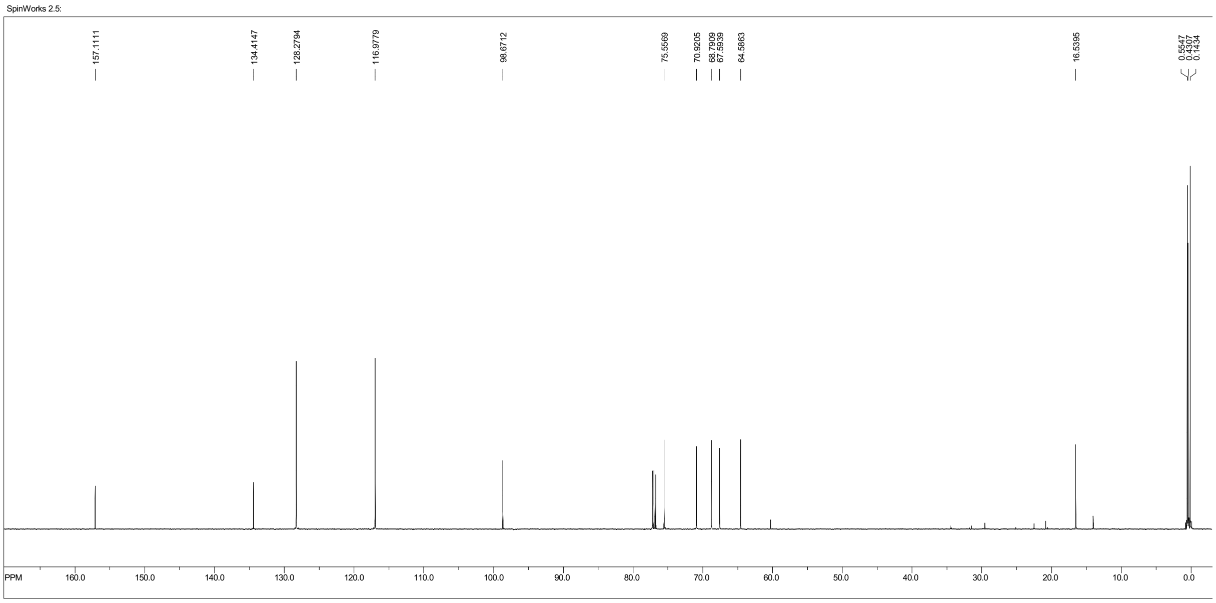

Fig. S20 ^{13}C NMR spectrum (125 MHz) of **4** in $CDCl_3$.

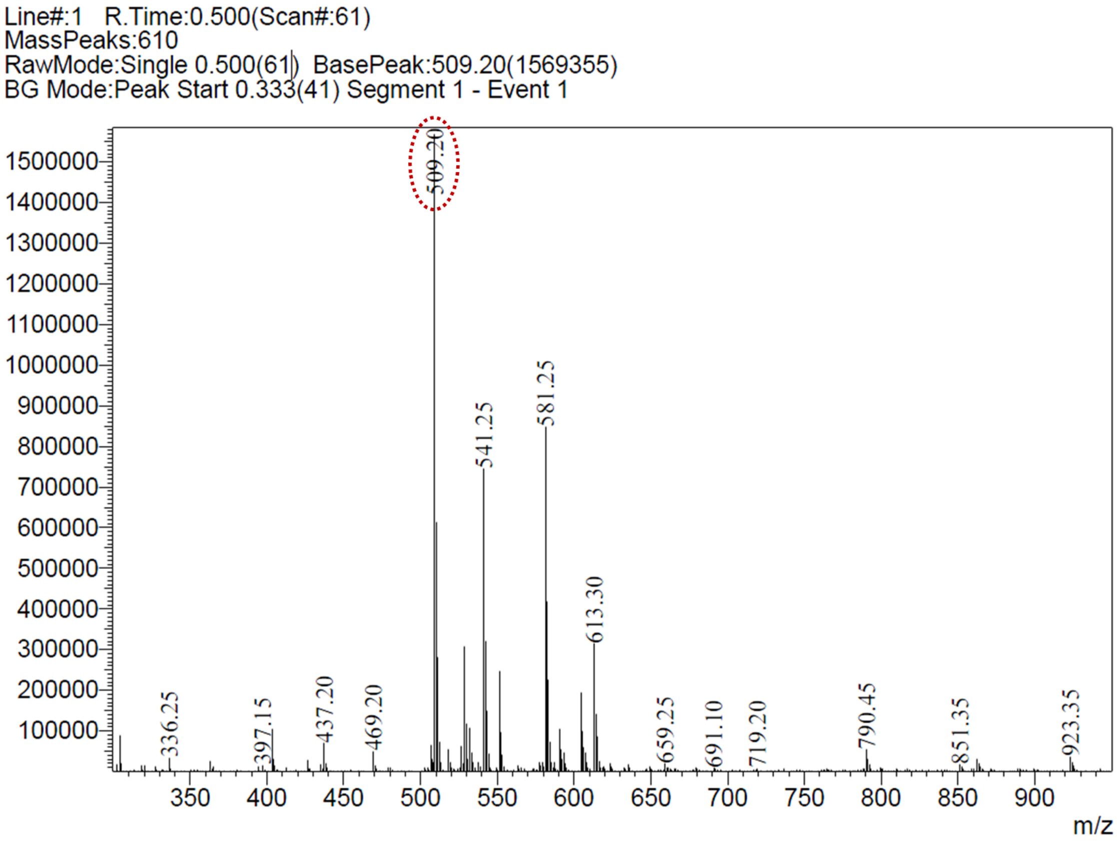

Fig. S21 ESI-MS spectrum of **4**.

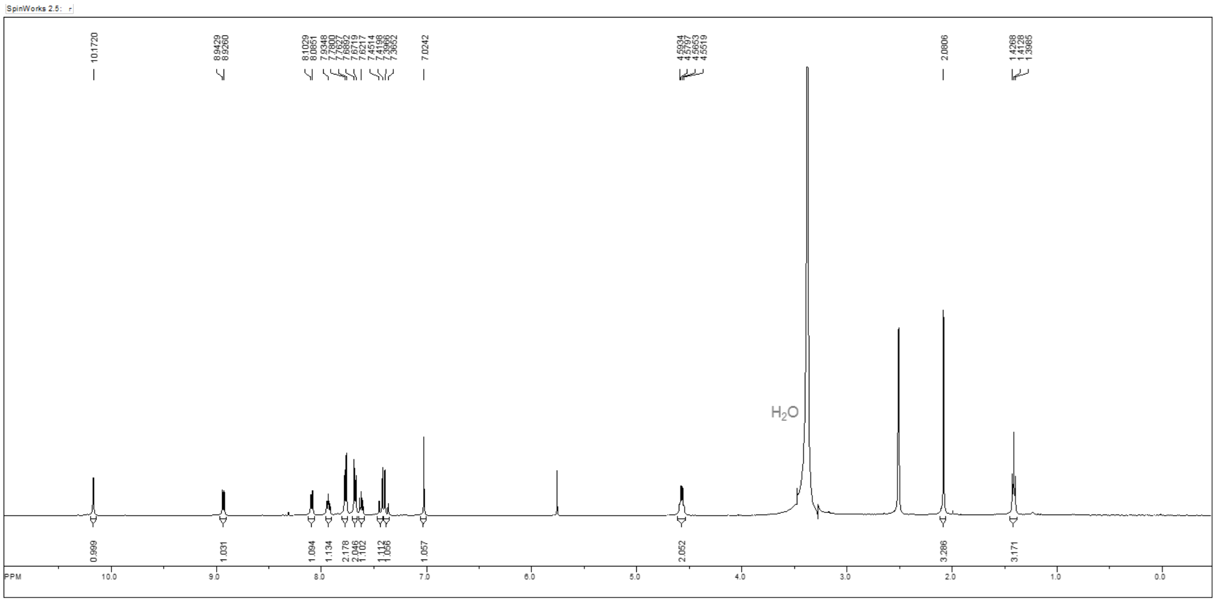

Fig. S22 ^{1}H NMR spectrum (500 MHz) of **2** in DMSO-d_6.

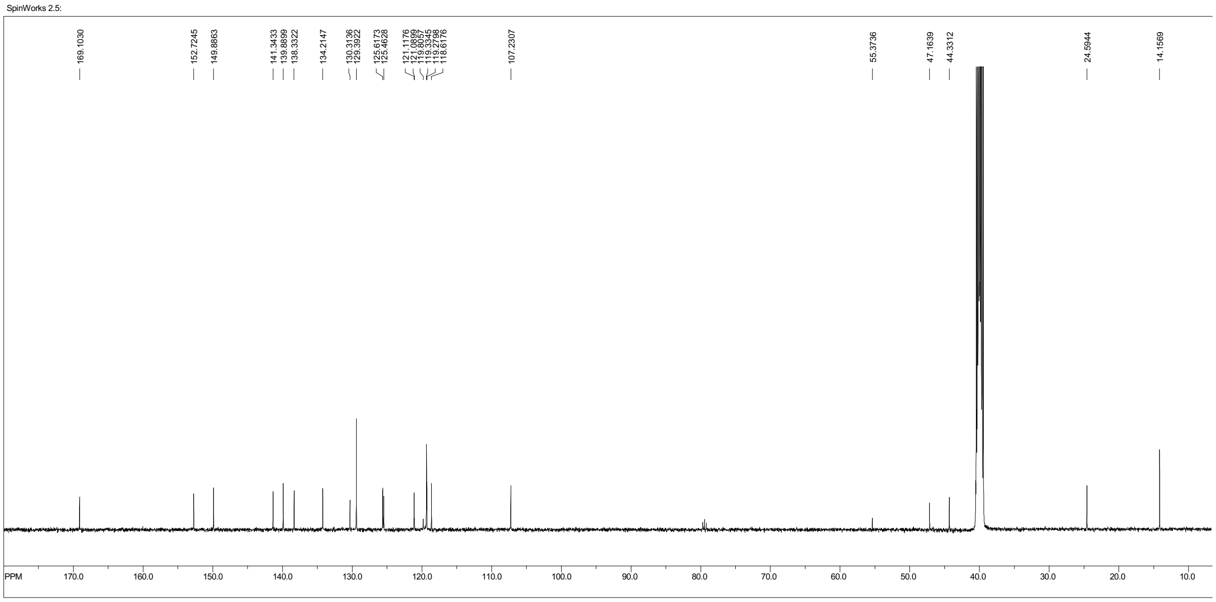

Fig. S23 ^{13}C NMR spectrum (125 MHz) of **2** in DMSO-d_6.

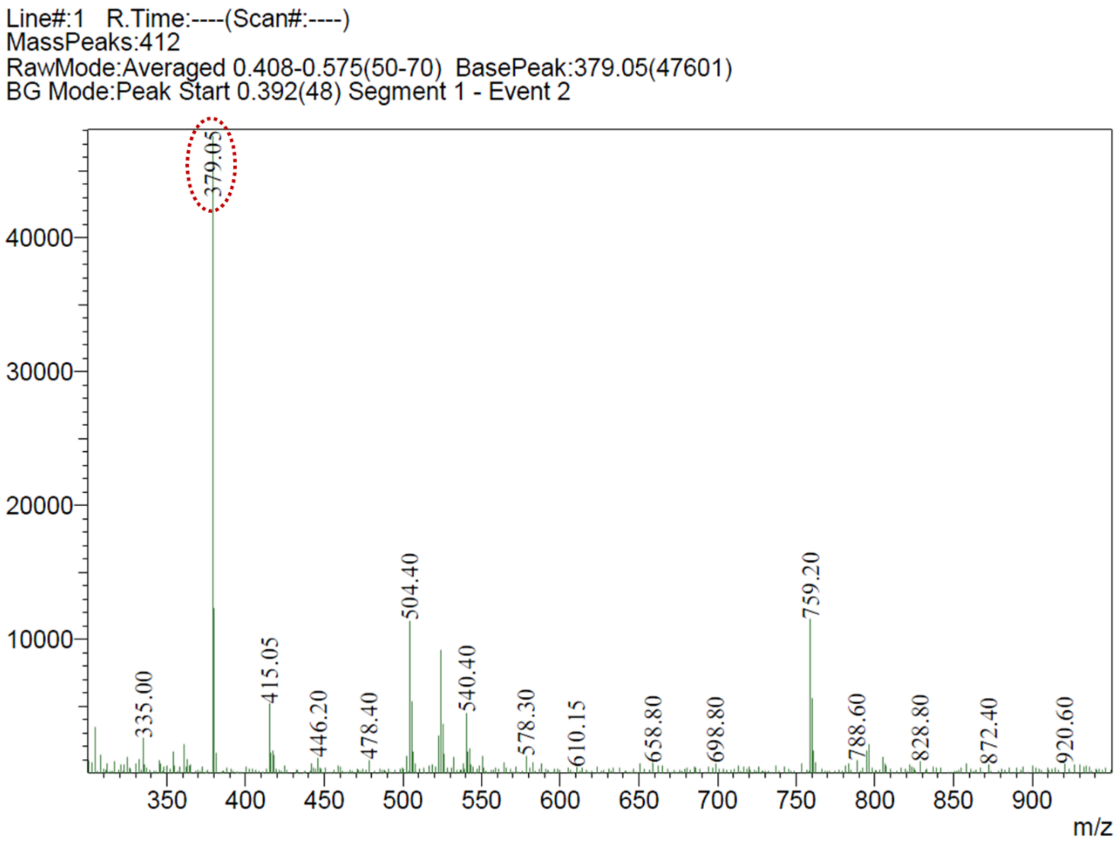

Fig. S24 ESI-MS spectrum of **2**.

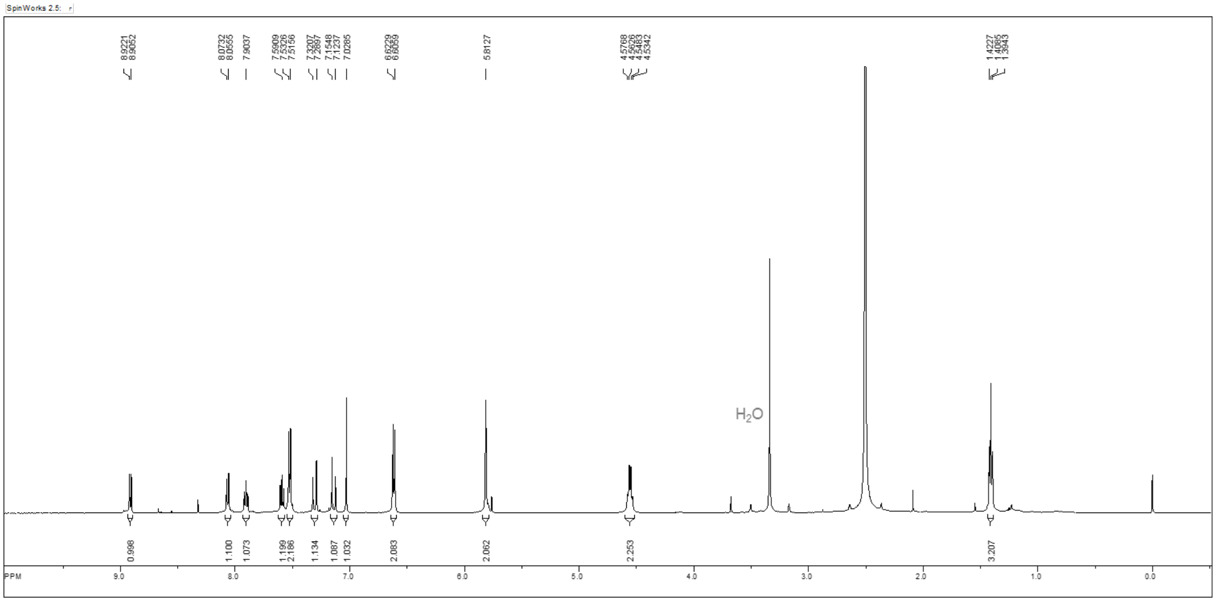

Fig. S25 ^{1}H NMR spectrum (500 MHz) of **QM-NH$_2$** in DMSO-d_6.

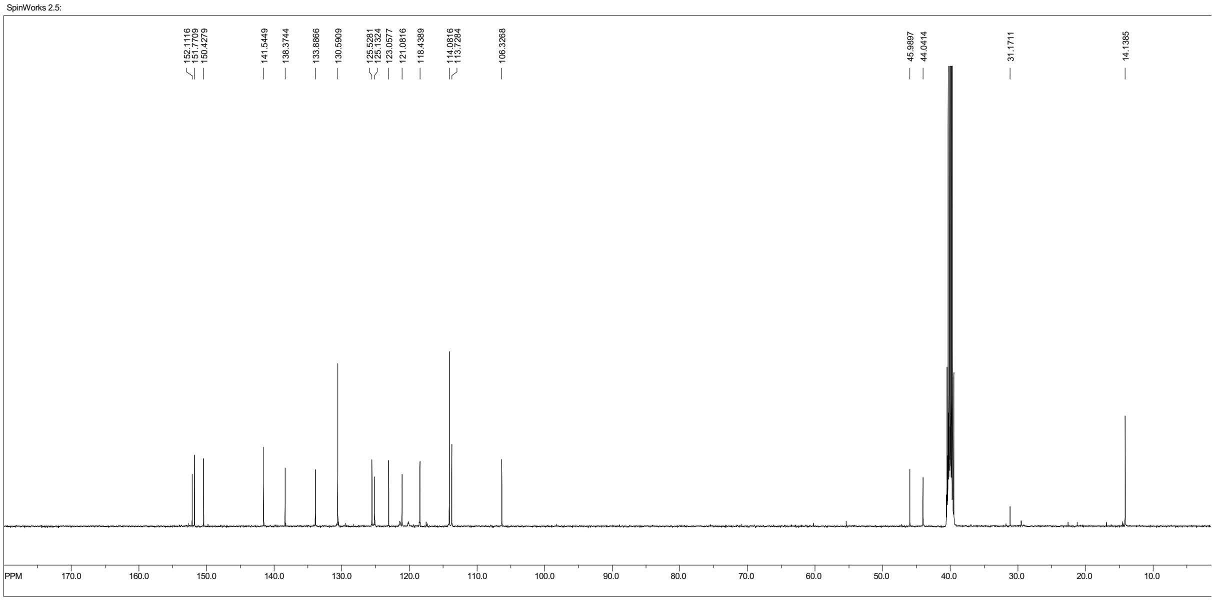

Fig. S26 ^{13}C NMR spectrum (125 MHz) of **QM-NH$_2$** in DMSO-d_6.

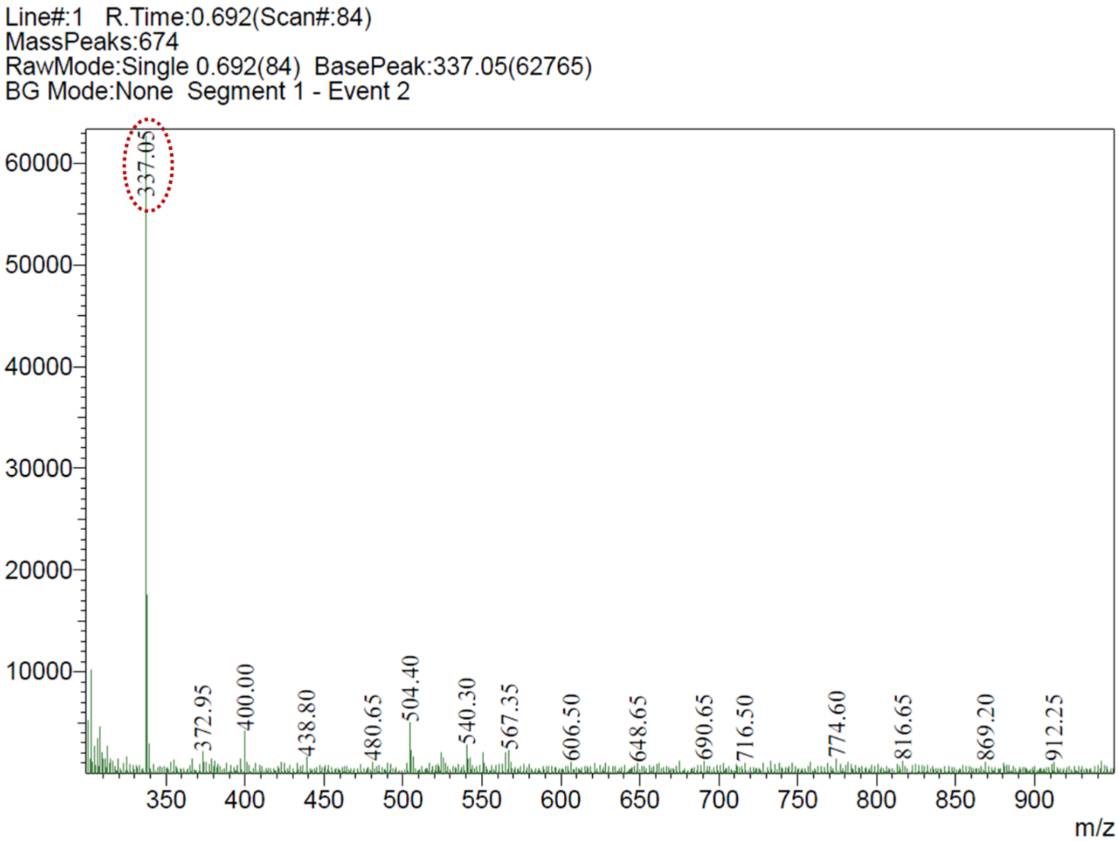

Fig. S27 ESI-MS spectrum of **QM-NH$_2$**.

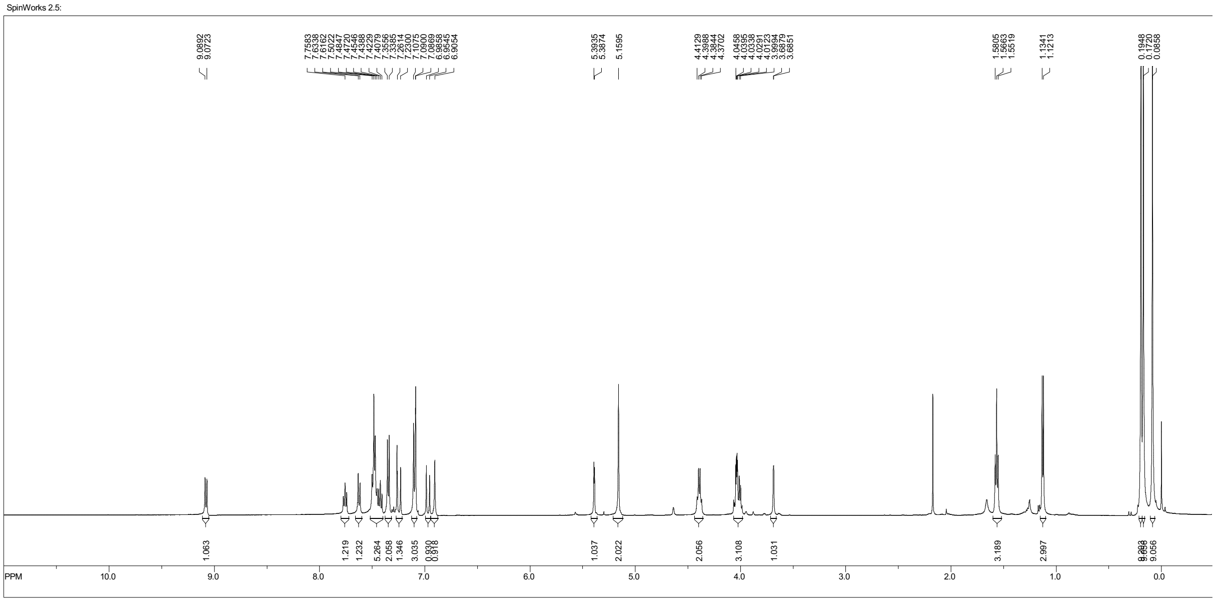

Fig. S28 ^{1}H NMR spectrum (500 MHz) of **1** in $CDCl_3$.

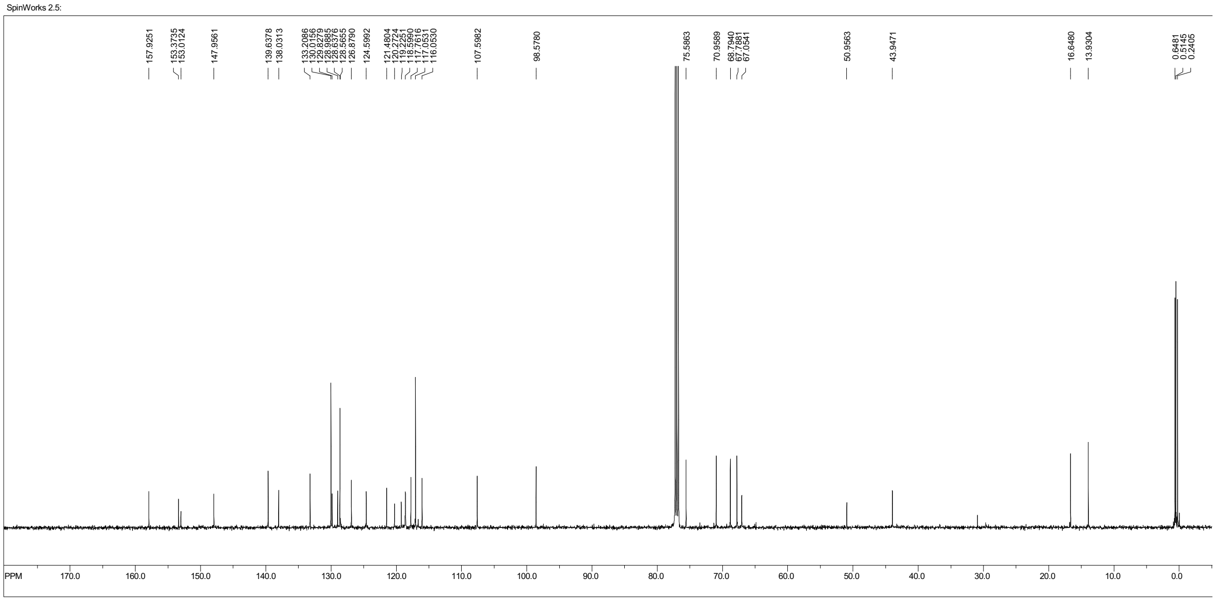

Fig. S29 ^{13}C NMR spectrum (125 MHz) of **1** in $CDCl_3$.

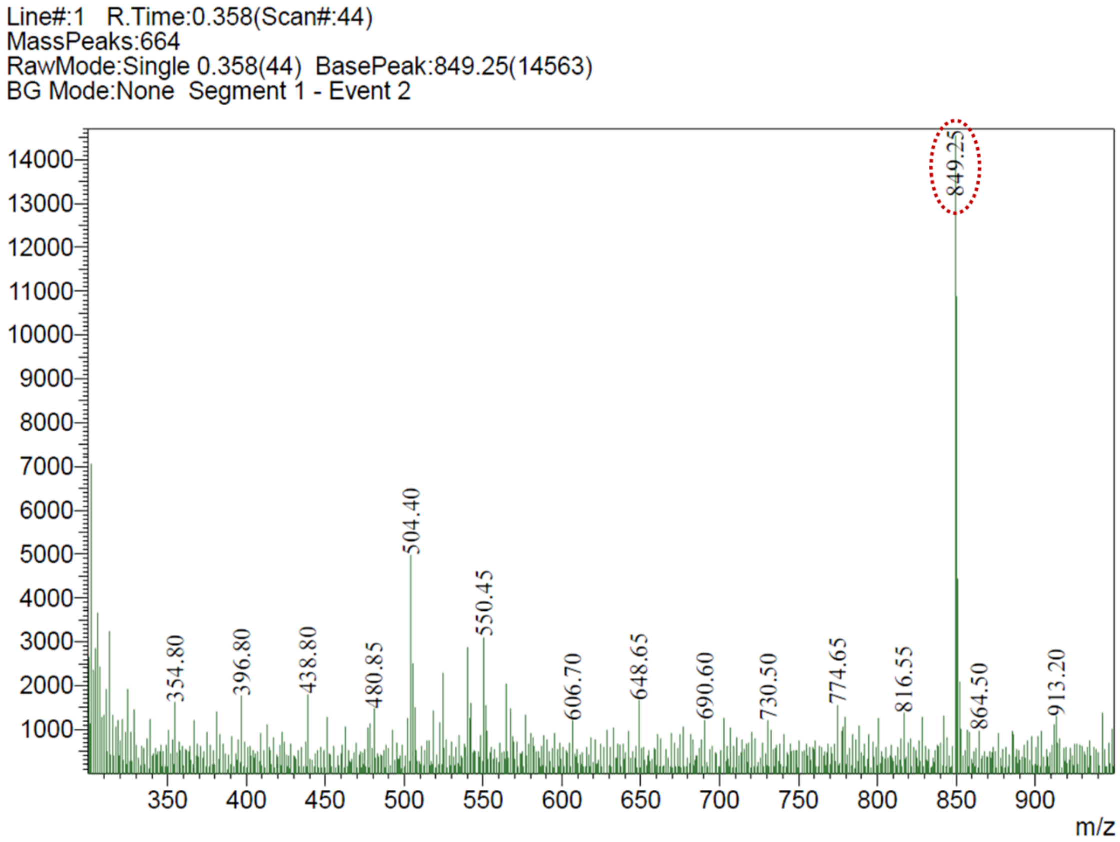

Fig. S30 ESI-MS spectrum of **1**.

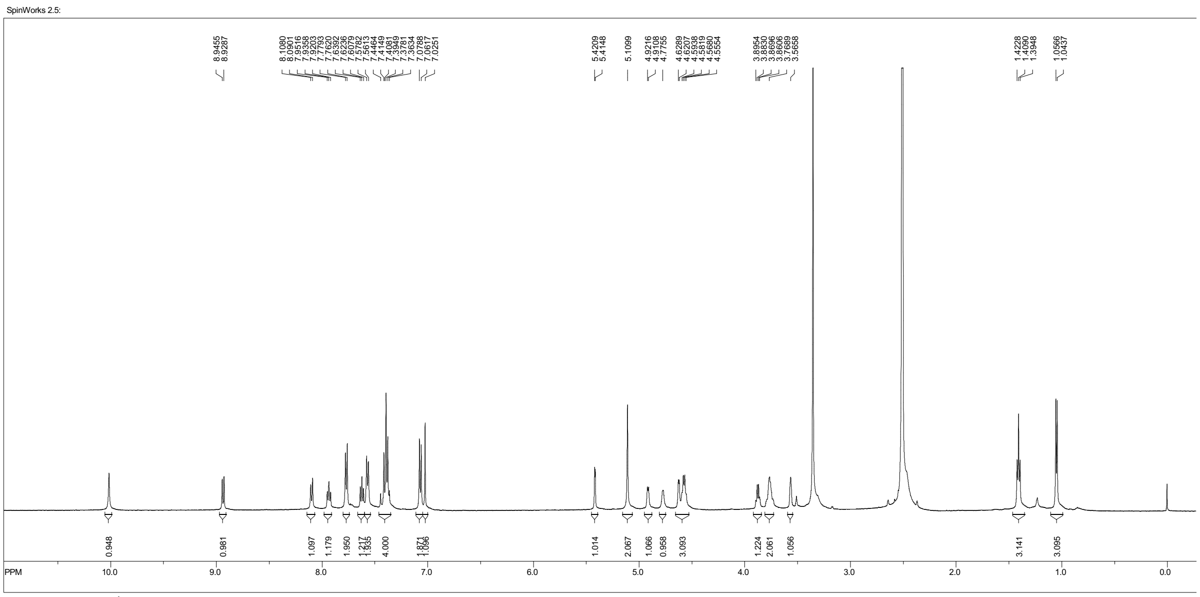

Fig. S31 ^{1}H NMR spectrum (500 MHz) of **QM-NHαfuc** in DMSO-d_6.

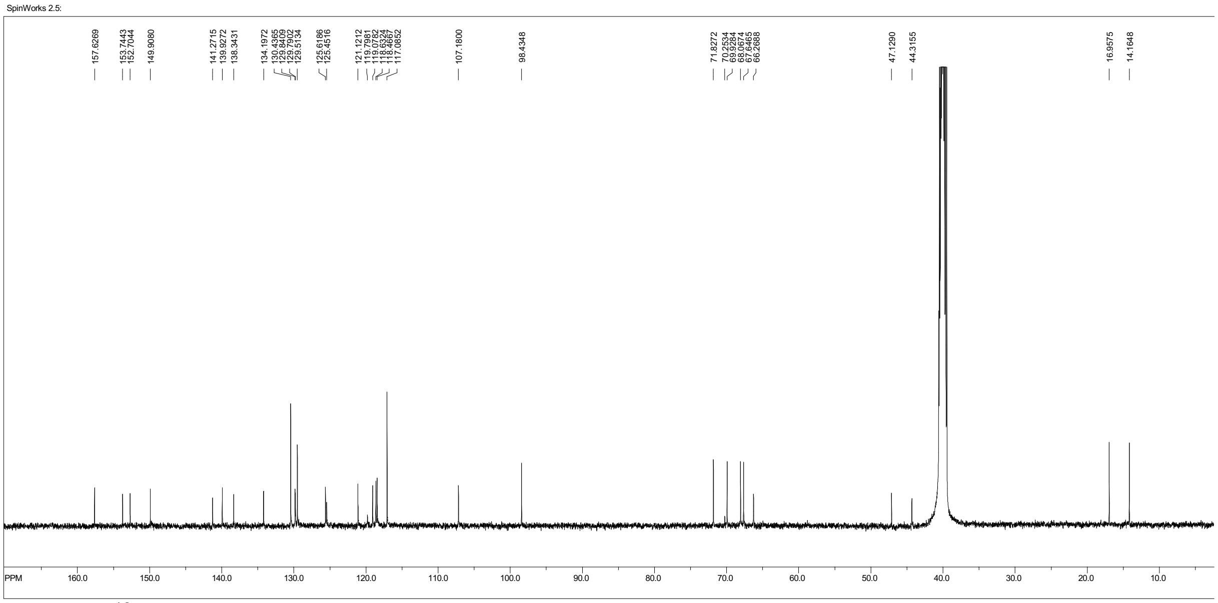

Fig. S32 ^{13}C NMR spectrum (125 MHz) of **QM-NHαfuc** in DMSO-d_6.

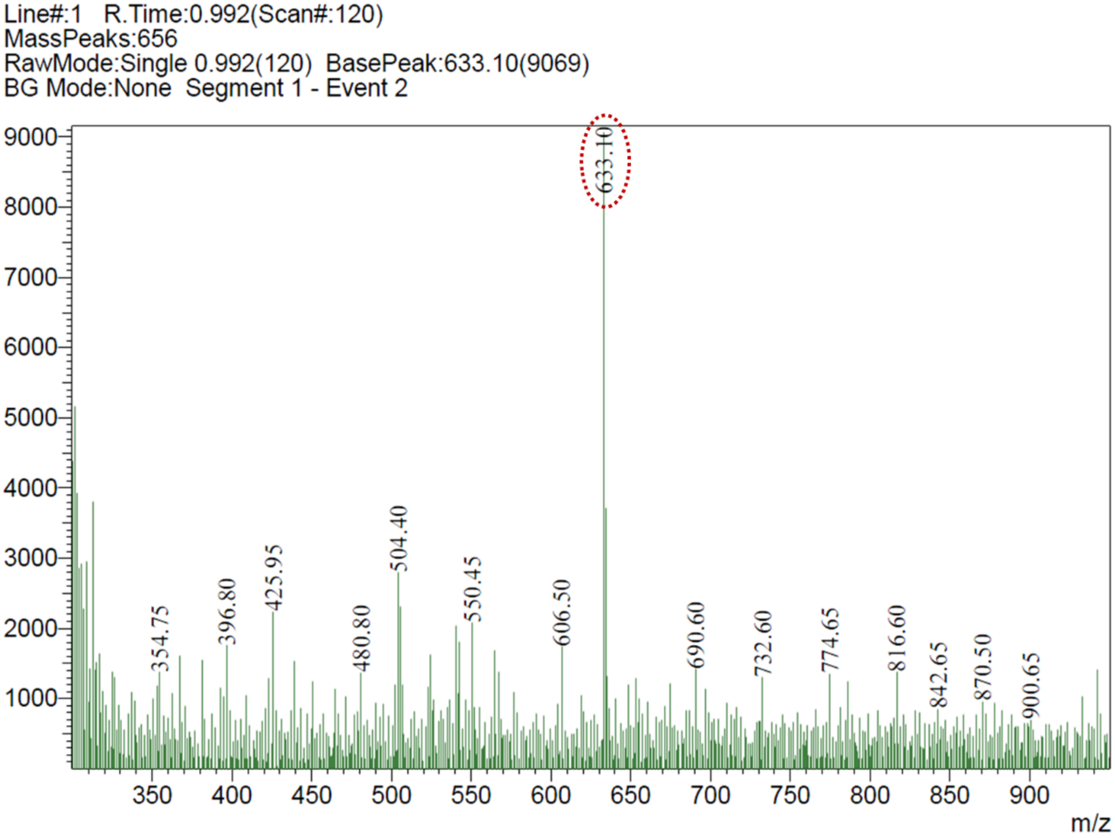

Fig. S33 ESI-MS spectrum of **QM-NHαfuc**.
